# Effects of Stand-Alone Digital Lifestyle Interventions on Weight-Related Outcomes in Adults With Overweight or Obesity: Systematic Review and Meta-Analysis of Randomized Controlled Trials

**DOI:** 10.2196/81070

**Published:** 2026-05-04

**Authors:** Si-An Lee, Jin-Hyuck Park

**Affiliations:** 1 Department of ICT convergence, The Graduate School, Soonchunhyang University, Asan, Republic of Korea; 2 Department of Occupational Therapy, College of Medical Science, Soonchunhyang University, Room 1401, Medical Science, 22, Soonchunhayng-ro, Shinchang-myeon, Asan, 31538, Republic of Korea, 01064362419

**Keywords:** digital intervention, digital device, lifestyle, weight, diet

## Abstract

**Background:**

Obesity is a major global health concern, and scalable digital solutions are urgently needed. While digital lifestyle interventions (DLSIs) have shown promise, prior meta-analyses often included hybrid formats with human support, limiting insights into the effectiveness of fully digital interventions.

**Objective:**

This study aimed to evaluate the independent effects of standalone DLSIs—defined as interventions delivered exclusively via digital platforms without in-person or adjunctive support—on anthropometric and dietary outcomes in adults with overweight or obesity.

**Methods:**

We searched MEDLINE, Embase, PsycINFO, Web of Science, and the Cochrane Library from inception through March 4, 2026. Eligible studies were randomized controlled trials (RCTs) evaluating stand-alone DLSIs in adults with overweight or obesity. Interventions were included if they targeted diet or physical activity exclusively through digital platforms. We included fully automated, asynchronous, or one-to-many synchronous systems without individualized support. Studies involving hybrid interventions, including one-to-one synchronous human interaction, nonadult populations, or non-RCT designs, were excluded. Two independent reviewers performed study selection and data extraction. Risk of bias was assessed using the Cochrane Risk of Bias 2.0 tool (Cochrane Bias Methods Group). Meta-analysis used a random-effects model with the Hartung-Knapp-Sidik-Jonkman method, and heterogeneity was assessed using *I*^2^ statistics. The certainty of evidence was evaluated using the Grading of Recommendations, Assessment, Development, and Evaluation approach.

**Results:**

A total of 19 RCTs involving 3556 participants were included. Stand-alone DLSIs significantly improved anthropometric outcomes compared to controls (standardized mean difference 0.26, 95% CI 0.14‐0.38; 95% prediction interval [PI] −0.16 to 0.68; *P*<.001; 19 studies; n=3556; *I*^2^=56.1%), corresponding to an additional weight loss ranging from 2.62 kg to 6.55 kg, depending on the baseline body weight. Significant improvements were also found in dietary outcomes (standardized mean difference 0.26, 95% CI 0.04‐0.48; 95% PI −0.29 to 0.81; *P*=.008; 8 studies; n=1365; *I*^2^=57.5%). Subgroup analyses for anthropometric outcomes revealed significant differences only by control group type (*P*<.001), with waitlist controls showing the largest effect. For dietary outcomes, no significant subgroup differences were found (*P*>.05). While most studies showed a low risk of bias, substantial statistical heterogeneity was observed in some outcomes. Consequently, the certainty of evidence for both outcomes was rated as moderate.

**Conclusions:**

This review is innovative as it is the first to isolate the pure efficacy of stand-alone DLSIs by excluding synchronous human support. Our findings provide moderate-certainty evidence that these tools are effective for weight management and dietary improvement without human intervention. While stand-alone DLSIs offer a highly scalable, cost-effective first-step intervention, the PIs included zero, and substantial heterogeneity was observed, suggesting that benefits may vary across settings. Future research should identify user characteristics that maximize engagement with unguided digital tools.

## Introduction

Obesity remains a critical global health challenge, with far-reaching implications for morbidity, mortality, and health care systems. According to the latest data from the World Health Organization (WHO), the global prevalence of obesity among adults aged 18 years and older has more than doubled, rising from 7% in 1990 to 16% in 2022 [1]. Recent evidence from 2023 further indicates that if current trends persist, over 4 billion people, more than half of the world’s population, will be living with overweight or obesity by 2035, placing an unprecedented economic burden on global health care infrastructures [2]. This escalating trend is projected to exert a staggering economic impact, with the global economic burden estimated to reach US $4.32 trillion annually by 2035, a figure comparable to the impact of the COVID-19 pandemic [2]. Overweight or obesity is a major risk factor for numerous noncommunicable diseases—including cardiovascular disease, type 2 diabetes, musculoskeletal disorders, and certain cancers—and contributes substantially to the global burden of disease and premature death [3]. This growing epidemic necessitates accessible, sustainable, and scalable strategies for weight management.

Lifestyle interventions, which typically integrate dietary modification, physical activity enhancement, and behavioral change techniques, have emerged as cornerstone strategies in the clinical management of overweight or obesity [4]. Traditionally, these interventions have been delivered through face-to-face counseling or group-based sessions and have demonstrated efficacy in reducing body weight and improving cardiometabolic outcomes [5]. Structured behavioral components, such as goal setting, self-monitoring, and problem solving, are widely recognized and incorporated into clinical practice guidelines for overweight or obesity treatment [6].

However, a key limitation of traditional lifestyle interventions is their reliance on in-person delivery, which poses considerable barriers to access. The requirement for physical attendance may hinder participation among individuals in rural or underserved communities, those with mobility or scheduling constraints, and those facing socioeconomic disadvantages [7]. These accessibility challenges were further exacerbated during the COVID-19 pandemic, which disrupted in-person care worldwide and underscored the urgent need for scalable alternatives [8]. In the postpandemic era, there has been a significant shift toward digital-first health care models, driven by the need for cost-effective solutions that can bypass the limitations of human-led medical resources [9].

Digital health technologies offer a compelling solution to these limitations. Digital lifestyle interventions (DLSIs)—including web-based platforms, mobile apps, and SMS text messaging–based systems—enable the delivery of personalized, interactive, and cost-effective behavioral support at scale [10]. These interventions can promote engagement through real-time feedback, self-monitoring tools, and flexible access, making them particularly well-suited for wide implementation across diverse populations [11]. Recent advancements in artificial intelligence (AI) and automated tailoring have further enhanced the potential of these platforms to provide high-quality, personalized support without the need for constant human supervision [10,12].

Prior systematic reviews have reported that digital interventions yield modest benefits over conventional offline approaches in facilitating weight loss [12,13]. However, these conclusions are often constrained by the inclusion of hybrid interventions that combine digital components with adjunctive modalities, such as web-based platforms supplemented by therapist support or face-to-face counseling. Such designs make it difficult to isolate the independent effect of digital platforms. Further complicating the evidence base, one meta-analysis included online interventions delivered in group formats—such as virtual group meetings—which, while not involving in-person contact, may introduce behavioral confounders, such as peer accountability and social facilitation [14]. Additionally, another review focused exclusively on web-based interventions, excluding alternative digital modalities such as mobile apps or SMS text messaging–based tools, and thereby failed to capture the broader spectrum of modern digital delivery systems [15].

Given the limitations of prior evidence syntheses—many of which have included hybrid or group-mediated interventions—there remains a critical need for a focused meta-analysis that isolates the independent impact of digital delivery. While hybrid models integrating in-person coaching have shown promise, their high operational costs and lack of scalability limit their utility for large-scale public health programs [16]. This creates a critical knowledge gap regarding the stand-alone efficacy of digital technology. By isolating digital interventions from individualized human-facilitated components, this systematic review and meta-analysis provides a clearer estimation of digital intervention effects. This methodological refinement eliminates confounding from human-facilitated components, allowing for a clearer assessment of the technology’s intrinsic impact. Furthermore, by synthesizing data across a wide range of delivery formats, including mobile apps, web platforms, and SMS text messaging–based systems, this study captures the contemporary landscape of digital health tools. This methodological focus allows for a more precise and high-certainty assessment of the independent effects of stand-alone DLSIs on weight management in adults with overweight or obesity.

Therefore, the primary objective of this study was to evaluate the efficacy of stand-alone DLSIs on anthropometric and dietary outcomes in adults with overweight or obesity. Specifically, we aimed to (1) quantify the pooled effect size of these interventions compared to various control conditions, (2) identify potential moderators of effectiveness through subgroup analyses, and (3) assess the certainty of the evidence to provide clinical and policy recommendations for scalable weight management.

## Methods

### Overview

This systematic review and meta-analysis follows the PRISMA (Preferred Reporting Items for Systematic Reviews and Meta-Analyses) guidelines and was prospectively registered with PROSPERO (CRD420251053974; Checklist 1). While the study was conducted according to the preregistered protocol, we additionally used the Hartung-Knapp-Sidik-Jonkman (HKSJ) method for the random-effects model and calculated 95% prediction intervals (PIs) to provide a more conservative and robust estimation of the evidence, which were not explicitly detailed in the initial protocol.

### Eligibility Criteria

Studies were included if they met the following criteria: (1) adults (≥18 years) with overweight or obesity (BMI≥23 kg/m^2^); (2) the intervention involved lifestyle interventions aimed at weight management, delivered exclusively via digital platforms or technologies, including mobile apps, computer software, websites, or SMS text messaging; (3) the intervention was delivered independently without any in-person human support. We defined stand-alone DLSIs as those where the core therapeutic components—including feedback, goal setting, and progress monitoring—were driven exclusively by digital platforms. Specifically, we included all digital interventions that did not require physical, face-to-face clinical contact, such as automated or periodic app notifications, SMS text messaging reminders, and preprogrammed, asynchronous feedback (eg, batch-sent educational emails or system-generated responses triggered by user-entered data), as these represent scalable alternatives to traditional resource-intensive programs. For studies using potentially ambiguous terminology (eg, “coaching,” “messaging,” or “peer support”), we verified the non–in-person nature of these components by cross-referencing study protocols or technical implementation sections. This verification ensured that any human involvement was neither in-person nor one-to-one support; (4) a non-DLSI control group was used for comparison, such as active controls, nonspecific active controls, or waitlist controls; (5) outcomes included anthropometric measures (eg, body weight, BMI, or waist circumference) or dietary measures (eg, caloric intake); and (6) the study design was a randomized controlled trial (RCT). Exclusion criteria were as follows: (1) participants were not primarily selected based on overweight or obesity status; (2) participants were pregnant women; (3) any intervention incorporating in-person human support (eg, face-to-face counseling or physical clinical visits) where the independent effect of the digital technology could not be isolated; (4) interventions involving resource-intensive, one-to-one digital interaction with human providers. Specifically, we excluded labor-intensive, real-time interaction such as personalized phone calls or individual video conferencing, as these do not align with the scalable nature of stand-alone DLSIs; (5) studies included DLSIs in the control group; and (6) studies lacking sufficient data for meta-analysis were excluded. For potentially eligible studies with incomplete or ambiguous data, we attempted to contact the corresponding authors to obtain the missing information. If no response was received or the provided data remained inadequate for calculating effect sizes, the studies were excluded from both the systematic review and the meta-analysis. Both peer-reviewed papers and conference abstracts were included in the main analysis, although conference abstracts were required to report RCT findings explicitly.

### Information Sources

The electronic databases were searched by the corresponding author using MEDLINE, Embase, PsycINFO, Web of Science, and the Cochrane Library to search for peer-reviewed papers in English, with no start date and no language restrictions. To ensure the specific indexing and search capabilities of each database were fully used, all databases were searched individually through their respective official interfaces rather than using a multidatabase search platform. We did not search study registries (eg, the WHO International Clinical Trials Registry Platform) for unpublished or ongoing trials, as our search was focused on peer-reviewed, published literature.

### Search Strategy

We conducted and reported our literature search in accordance with the PRISMA-S (Preferred Reporting Items for Systematic Reviews and Meta-Analyses–Search Extension) guidelines to ensure transparency and reproducibility [17] (Checklist 2). The search strategies were developed independently for this study and did not adopt or reuse search strings from prior literature reviews. The strategies were constructed by combining 3 main domains (digital interventions, obesity or overweight, and weight management) using a comprehensive set of Medical Subject Headings and relevant keywords tailored to each database’s indexing structure [15]. To ensure transparency and minimize the risk of missing studies, we followed the Cochrane Handbook guidelines for search strategy development. The full, unabridged search strategy for each database is provided in Multimedia Appendix 1. However, the search strategy did not undergo a formal peer review process by an external librarian.

The reference lists of all included studies and relevant studies were searched manually, and the gray literature was also searched by entering the same keywords into Google on May 18, 2025. During the preparation and revision of the meta-analysis, the search was repeated 4 times to identify newly published trials. The last search was conducted on March 4, 2026, following the previous update on June 30, 2025.

### Selection Process

After eliminating duplicate records, a 2-phase screening procedure was implemented: initial screening of titles and abstracts, followed by a full-text review. Two independent reviewers (SAL and JHP) assessed the eligibility of each record at both stages using Covidence (Veritas Health Innovation). To maintain consistency across the process, a random 10% subset of entries was jointly reviewed during each screening phase to calculate interrater agreement. Any disagreements were resolved through discussion and consensus between the 2 reviewers until a mutual agreement was reached. Interrater reliability, quantified using Cohen kappa, indicated near-perfect agreement at both the title and abstract screening stage (0.92) and full-text review (0.94). Reviewers remained blinded to each other’s assessments throughout to ensure objectivity. After completing the literature search, 2 independent reviewers screened the titles and abstracts of all retrieved records to assess eligibility. Full-text papers were subsequently obtained for trials deemed potentially relevant and reviewed in detail for inclusion. In addition, the reference lists of all included papers and related review papers were manually examined to identify any supplementary studies.

### Data Collection

Data were extracted from each included study by 2 independent reviewers (SAL and JHP) using a standardized, pilot-tested data extraction form to ensure accuracy and consistency. Any discrepancies in the extracted data were resolved through detailed discussion and internal cross-checking between the 2 reviewers. If essential data were missing or unclear in the original reports, we attempted to contact the primary authors via email to obtain the necessary information.

### Data Items

From each included study, the following information was extracted when available: (1) bibliographical data (ie, first author and year); (2) sample characteristics (ie, sample size, mean age, and population); (3) intervention characteristics (ie, details of intervention and control conditions, target lifestyle, theoretical framework, delivery format, duration, and outcome domains); (4) participant retention rates, defined as the proportion of participants in the intervention group who completed the postintervention assessment relative to the total number of participants initially randomized; and (5) data required to calculate within-group or between-group effect sizes for RCTs. To calculate effect sizes, both baseline (preintervention) and postintervention data were extracted for all outcomes. However, follow-up data were excluded from the meta-analysis to maintain consistency across studies and avoid bias resulting from varying follow-up durations.

Eligible interventions were defined as those aiming to change diet and/or physical activity behaviors using established behavior change techniques (BCTs; eg, goal setting and self-monitoring). Interventions were included if at least 50% of their content targeted behavior change in either domain and incorporated one or more BCTs listed in the BCT Taxonomy v1 [18]. Detailed BCT classification for each included study is provided in Multimedia Appendix 2. The primary outcome was anthropometric measures, including body weight, BMI, and waist circumference. When a study reported multiple anthropometric variables, we applied a predefined hierarchy to select a single outcome for the primary analysis: (1) body weight, (2) BMI, and (3) waist circumference. Secondary outcomes included dietary measures, with total energy intake (kcal/d) prioritized as the primary dietary metric.

### Study Risk of Bias Assessment

Risk of bias in individual studies was appraised independently by 2 independent reviewers using the Cochrane Collaboration’s Risk of Bias 2 tool guidelines in 5 domains: randomization process, deviations from intended interventions, missing outcome data, measurement of the outcome, and selection of the reported result [19]. A study was categorized as high risk if at least one domain was rated “high risk.” If there were no high-risk domains but at least one domain raised concerns, the study was rated as “some concerns.” Only studies with low risk in all domains were designated as “low risk.”

### Effect Measures

The primary outcomes (anthropometric measures) and secondary outcomes (dietary measures) were continuous variables. To account for the diversity in measurement scales across studies, standardized mean differences (SMDs) with 95% CIs were used as the summary effect measure. SMD estimates of 0.20, 0.50, and 0.80 were interpreted as small, moderate, and large effect sizes, respectively [20]. For outcomes where a lower value indicates clinical improvement (eg, body weight, BMI, and waist circumference), the direction of the effect size was aligned during data coding in the Comprehensive Meta-Analysis software (Biostat) so that positive SMDs consistently represent a greater reduction in the intervention group. To avoid double-counting in the meta-analysis and ensure that each study contributed independent participants, we only included one pair of baseline and postintervention data per study in the primary analysis, selected according to the aforementioned hierarchy. SMDs were calculated using the change from baseline to postintervention for both the intervention and control groups. In cases where a study reported outcomes for multiple intervention arms compared to a single control group, we combined the intervention groups or split the control group to maintain the independence of observations. When change scores were not directly reported, they were calculated using baseline and postintervention means and SDs, assuming a correlation coefficient of 0.5 where necessary.

### Synthesis Methods

Quantitative synthesis was performed using a random-effects model, as clinical and methodological heterogeneity was expected across the included stand-alone digital interventions. To improve the precision of the pooled estimates and minimize the risk of false positives, the HKSJ method was applied.

Statistical heterogeneity was assessed using the Cochran Q statistic and the *I*^2^ index, with *I*^2^>50% indicating substantial heterogeneity [21]. To further quantify the dispersion of effects in real-world settings, 95% PIs were calculated alongside the pooled estimates. Subgroup analyses were prespecified based on the type of control group (active vs waitlist), intervention duration (≤12 weeks vs >12 weeks), delivery type (app vs website), and target lifestyle (diet vs physical activity vs combined). All statistical analyses were conducted using Comprehensive Meta-Analysis (version 3) software (Biostat) and R (version 4.5.1; R Foundation for Statistical Computing) with the “*meta*” package.

### Reporting Bias Assessment

To assess potential reporting biases, including publication bias and small-study effects, we used both visual and statistical methods. Funnel plot asymmetry was visually inspected. Additionally, the Egger regression test was performed to statistically evaluate small-study effects, with a *P*<.10 indicating significant asymmetry [22]. Furthermore, a trim-and-fill sensitivity analysis was conducted to estimate the potential impact of missing studies on the pooled effect size [23]. We also considered whether the observed asymmetry could be attributed to factors other than publication bias, such as differences in study quality or substantial interstudy heterogeneity.

### Certainty Assessment

The Grading of Recommendations, Assessment, Development, and Evaluation framework was used to evaluate the certainty of evidence across 5 domains: risk of bias, imprecision, inconsistency, indirectness, and publication bias. Depending on the quality assessment within these domains, the certainty of evidence could be either downgraded or upgraded. The final classification for each outcome was assigned to 1 of 4 levels: “very low,” “low,” “moderate,” or “high” [24]. Two independent reviewers (SAL and JHP) assessed the evidence across 5 domains. Any discrepancies in the grading were resolved through consensus between the 2 reviewers.

### Ethical Considerations

This study is a systematic review and meta-analysis of previously published literature and did not involve direct interaction with human participants or the collection of identifiable private information. According to institutional and national guidelines (eg, the Bioethics and Safety Act of the Republic of Korea), this type of research is exempt from Institutional Review Board review as it uses publicly available data and carries no risk to individuals.

## Results

### Study Selection

**Figure 1.**
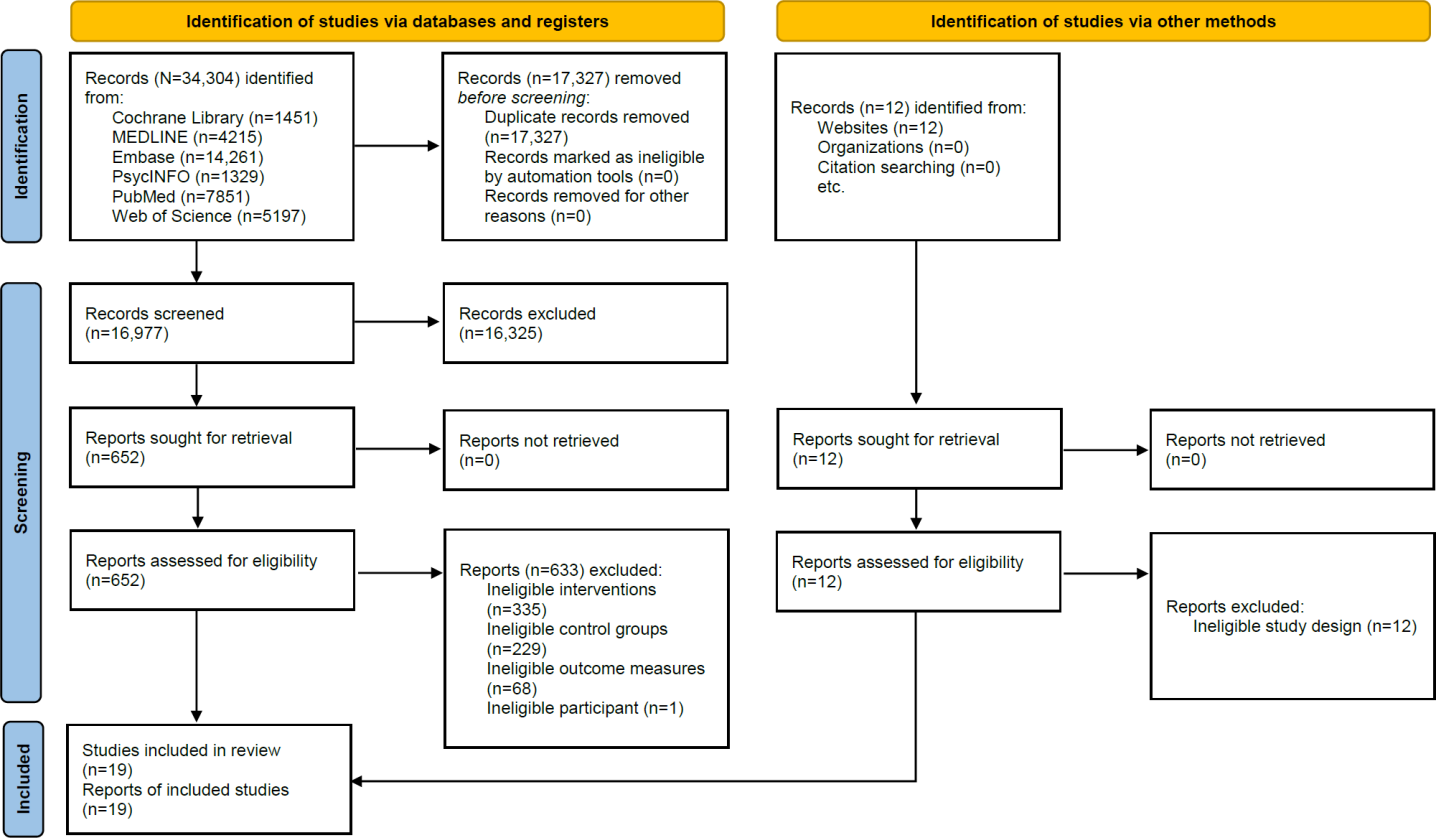
PRISMA (Preferred Reporting Items for Systematic Reviews and Meta-Analyses) flow diagram of included studies. The diagram displays the number of records identified, screened, assessed for eligibility, and included in the systematic review and meta-analysis.

A total of 34,304 literature records were identified from the databases, and 12 additional records were identified through other sources. After removing 17,327 duplicate records, 16,977 records were screened based on titles and abstracts, of which 16,325 were excluded. The primary reasons for exclusion at this stage were (1) clearly irrelevant topics (eg, nondigital clinical treatments or pharmacological interventions), (2) ineligible study types (eg, study protocols, review papers, or editorials), and (3) ineligible populations (eg, pediatric or adolescent samples) identifiable directly from titles or abstracts. Subsequently, 652 full-text papers were assessed for eligibility. Of these, 633 papers were excluded for the following reasons: ineligible combined interventions (n=335), ineligible control groups including digital interventions (n=229), lack of weight-related outcome measures (n=68), and ineligible participants (n=1). Finally, 19 papers were included in this systematic review and meta-analysis (Figure 1).

### Study Characteristics

Table 1 provides characteristics of the 19 included studies. All studies were published between 2007 and 2024. A total of 3556 participants (71% female) were included in the 19 trials, and the total sample sizes per study ranged from 31 to 650 participants. The mean participant age ranged from 29.4 to 51.2 years. All participants were classified as having overweight or obesity (BMI≥23 kg/m^2^), with varying inclusion thresholds across studies.

**Table 1.** Characteristics of the included studies in the meta-analysis.

Authors	Sample size	Mean age (SD) years	Female participants, n (%)	Population (BMI)	Target lifestyle	Behavioral strategy	Delivery format	Comparison	Duration
Allen et al [25]	E^a^=17 and C^b^=18	43.9 (12.6)	29 (82.8)	28‐42	Diet and physical activity	Self-monitoring with calorie targets and behavioral counseling for diet and physical activity.	App	Active control: in-person nutritional counseling	12 weeks
Brame et al [26]	E=78 and C=75	50.0 (10.8)	70 (45.7)	27.5‐34.9	Diet	Self-monitoring and personalized goal setting for diet and weight with structured coaching and feedback.	Website	Nonspecific active control (usual care)	12 weeks
Carter et al [27]	E1=43, E2=43, and C=43	41.8 (9.1)	99 (76.7)	≥27	Diet	Self-monitoring of diet and activity with calorie goals, feedback, and tailored text support.	E1: App E2: Website	Active control (food diary)	6 months
Chung et al [28]	E=19, C1=16, and C2=19	Not reported	34 (62.9)	≥25	Diet	Self-monitoring of dietary intake with personalized feedback based on nutrient analysis.	Website	C1: active control (food diary) and C2: waitlist control	12 weeks
Collins et al [29]	E=99 and C=104	41.8 (10.1)	118 (58.1)	25‐40	Diet and physical activity	Self-monitoring with tailored calorie targets, behavioral feedback for diet, and physical activity.	Website	Waitlist control	12 weeks
Dunn et al [30]	E=42 and C=38	47.9 (10.3)	Not reported	≥25	Diet and physical activity	Structured goal tracking and coaching for diet and physical activity.	Website	Waitlist control	15 weeks
Hurkmans et al [31]	E=30 and C=22	44.5 (11.3)	30 (57.6)	29‐34	Diet and physical activity	Self-monitoring and education for diet and physical activity.	App	Waitlist control	12 weeks
Kohl et al [32]	E=78 and C=75	48.9 (11.1)	109 (71.2)	27.5‐34.9	Diet	Self-monitoring with personalized diet logging, energy-density feedback, and interactive behavior-change activities.	Website	Nonspecific active control (education)	12 weeks
Kraschnewski et al [33]	E=50 and C=50	50.3 (10.9)	69 (69.0)	≥25	Diet and physical activity	Self-monitoring with goal setting and tailored behavior modeling for diet, activity, and weight control.	Website	Waitlist control	12 weeks
Krukowski et al [34]	E=161 and C=157	46.6 (10.0)	295 (92.7)	25‐50	Diet and physical activity	Self-monitoring with behavioral counseling for diet, physical activity, and weight control.	Website	Active control (in-person lifestyle intervention)	6 months
Lim et al [35]	E=99 and C=105	51.2 (9.7)	72 (35.2)	≥23	Diet and physical activity	Self-monitoring with feedback, goal setting, and dietitian messaging for diet, activity, and weight loss.	App	Waitlist control	6 months
Lugones-Sanchez et al [36]	E=318 and C=332	48.3 (9.6)	445 (68.4)	27.5‐40	Diet and physical activity	Self-monitoring with calorie feedback, activity tracking, and behavioral goal setting for diet and physical activity.	App	Active control (in-person counseling)	12 weeks
McConnon et al [37]	E=111 and C=110	47.7 (not reported)	170 (77.0)	≥30	Diet and physical activity	Self-monitoring with personalized advice, motivational feedback, and behavioral tools for diet and physical activity.	Website	Nonspecific active control (usual care)	12 months
Moravcova et al [38]	E=50 and C=50	43.3 (9.5)	71 (71.0)	≥30	Diet and physical activity	Self-monitoring with personalized daily goals, education, and dietitian support for diet, activity, and lifestyle behaviors.	App	Active control (in-person lifestyle intervention)	12 weeks
Padwal et al [39]	E=225, C1=215, and C2=211	40.4 (9.8)	540 (82.9)	≥35	Diet and physical activity	Self-guided behavior change program with modular content for diet and physical activity.	Website	C1: active control (in-person) and C2: nonspecific active control (education)	12 weeks
Steinberg et al [40]	E=47 and C=44	43.8 (11.0)	68 (74.7)	25‐40	Diet and physical activity	Self-monitoring with smart scale, weight tracking, and weekly tailored feedback with behavioral lessons.	Website	Waitlist control	6 months
Svetkey et al [41]	E=120 and C=123	29.4 (4.3)	169 (69.5)	≥25	Diet and physical activity	Self-monitoring with goal setting, behavioral prompts, and peer support for diet, activity, and weight management.	App	Waitlist control	24 months
Vaz et al [42]	E=15 and C=16	43.2 (3.5)	Not reported	25‐42	Diet and physical activity	Self-monitoring with wearable tracking, food photo logging, and coaching and peer feedback for diet, activity, and weight loss.	App	Waitlist control	6 months
Yardley et al [43]	E=45 and C=43	50.5 (13.5)	60 (68.1)	≥30	Diet and physical activity	Self-monitoring with goal setting, dietary choice, and cognitive-behavioral tools for weight management.	Website	Nonspecific active control (usual care)	6 months

a E: experimental group.

b C: control group.

The interventions targeted either diet alone or a combination of diet and physical activity [25-43]; notably, no study targeted physical activity alone. Common intervention strategies included self-monitoring, personalized goal setting, and tailored feedback. Delivery platforms included mobile apps (n=7) [25,31,35,36,38,41,42], websites (n=11) [26,28-30,32-34,37,39,40,43], or both (n=1) [27]. Comparison groups included active controls (eg, in-person nutritional counseling and food diaries; n=7) [25,27,28,34,36,38,39], nonspecific active controls (eg, education or usual care; n=5) [26,32,37,39,43], and waitlist controls (n=9) [28-31,33,35,40-42]. The duration of interventions varied from 12 weeks to 24 months, with 12-week programs being the most common (n=9; Table 1) [25,26,28,29,31-33,36,38,39]. To ensure the stand-alone nature of the included interventions, we meticulously verified the delivery mechanisms of all digital interactions. While some studies used terms such as coaching, feedback, or peer support, these were confirmed to be delivered through automated, system-generated algorithms or asynchronous, preprogrammed modules without real-time human clinical labor (Multimedia Appendix 3).

Body weight and BMI were the most frequently reported as primary outcomes. Eight studies also assessed dietary outcomes such as caloric intake and macronutrient composition [25,30-33,35,36,40]. Retention rates, as reported in Table 2, ranged from 44.1% to 95%, with most studies achieving rates above 70%. Mobile app interventions tended to show slightly higher retention compared to web-only platforms, although variation existed (Table 2).

**Table 2.** Adherence and measures characteristics. Retention rate (%) = (number of participants in the intervention group who completed the final follow-up / number of participants initially randomized to the intervention group) ×100.

Author, year	Retention rate, n/N (%)	Anthropometric measures	Dietary measures	Timing of measures
Allen et al [25]	10/17 (58.8%)	Body weight (kg), BMI (kg/m^2^), and waist circumference (cm)	Dietary, calories, fruit and vegetable, and sodium intake (Kcal/d)	Baseline and 12 weeks
Brame et al [26]	39/78 (50%)	Body weight (kg), BMI (kg/m^2^), and waist circumference (cm)	Not reported	Baseline and 12 weeks
Carter et al [27]	App: 40/43 (93%) Website: 19/43 (44.1%)	Body weight (kg), BMI (kg/m^2^), and body fat (%)	Not reported	Baseline, 6 weeks, and 6 months
Chung et al [28]	19/20 (95%)	Body weight (kg), BMI (kg/m^2^), body fat (%), and waist-to-hip ratio (%)	Not reported	Baseline, 6 weeks, and 12 weeks
Collins et al [29]	74/99 (74.7%)	Body weight (kg), BMI (kg/m^2^), and waist circumference (cm)	Not reported	Baseline and 12 weeks
Dunn et al [30]	(28/44) (66.6%)	Body weight (kg) and BMI (kg/m^2^)	Eating confidence (5-point Likert scale)	Baseline and 15 weeks
Hurkmans et al [31]	24/30 (80%)	BMI (kg/m^2^)	Energy intake (Kcal/d)	Baseline and 12 weeks
Kohl et al [32]	55/78 (70.5%)	Body weight (kg), fat mass (kg), fat-free mass (kg), and waist circumference (cm)	Energy and protein intake (Kcal/d), carbohydrate, fat, alcohol, and fiber intake (g/d)	Baseline, 12 weeks, 6 months, and 12 months
Kraschnewski et al [33]	43/50 (86%)	Body weight (kg) and BMI (kg/m^2^)	Caloric intake (Kcal/d)	Baseline and 12 weeks
Krukowski et al [34]	153/161 (95%)	BMI (kg/m^2^)	Not reported	Baseline and 6 months
Lim et al [35]	94/99 (94.9%)	Body weight (kg) and BMI (kg/m^2^)	Caloric intake (Kcal/d)	Baseline, 3 months, and 6 months
Lugones-Sanchez et al [36]	218/318 (67.9%)	Body weight (kg), BMI (kg/m^2^), waist circumference (cm), and hip circumference (cm)	Energy intake (Kcal/d)	Baseline, 3 months, and 12 months
McConnon et al [37]	54/111 (48.6%)	Body weight (kg)	Not reported	Baseline, 6 months, and 12 months
Moravcova et al [38]	32/50 (64%)	Body weight (kg), BMI (kg/m^2^), waist circumference (cm), body fat (%), and muscle mass (kg)	Not reported	Baseline, 3 months, and 6 months
Padwal et al [39]	166/225 (73.7%)	Body weight (kg) and BMI (kg/m^2^)	Not reported	Baseline and 12 weeks
Steinberg et al [40]	45/47 (95.7%)	Body weight (kg)	Caloric intake (Kcal/d)	Baseline, 3 months, and 6 months
Svetkey et al [41]	104/120 (86.6%)	Body weight (kg)	Not reported	Baseline, 6 months, 12 months, and 24 months
Vaz et al [42]	13/15 (86.6%)	Body weight (kg)	Not reported	Baseline and 6 months
Yardley et al [43]	39/45 (86.6%)	Body weight (kg)	Not reported	Baseline, 6 months, and 12 months

### Risk of Bias in Studies

**Figure 2.**
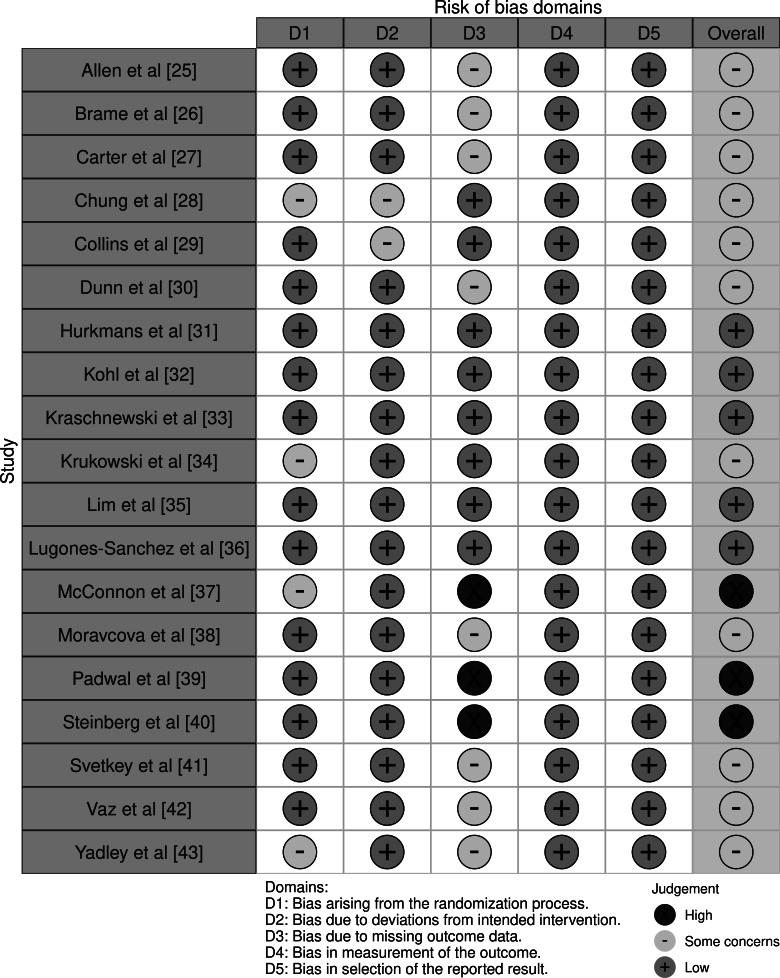
Risk of bias summary for included studies. This figure displays the overall risk of bias assessments across all included studies using the Cochrane Risk of Bias tool (version 2) [25-43].

Among the 19 studies, 3 studies were classified to have a high risk of bias, primarily due to issues in missing outcome data and selection of the reported results. The remaining 16 studies were judged as either low risk (n=7) or raising some concerns (n=9), with the most common concerns arising from deviations from intended interventions or lack of information on prespecified analysis plans (Figure 2 [25-43]).

### Results of Individual Studies

**Figure 3.**
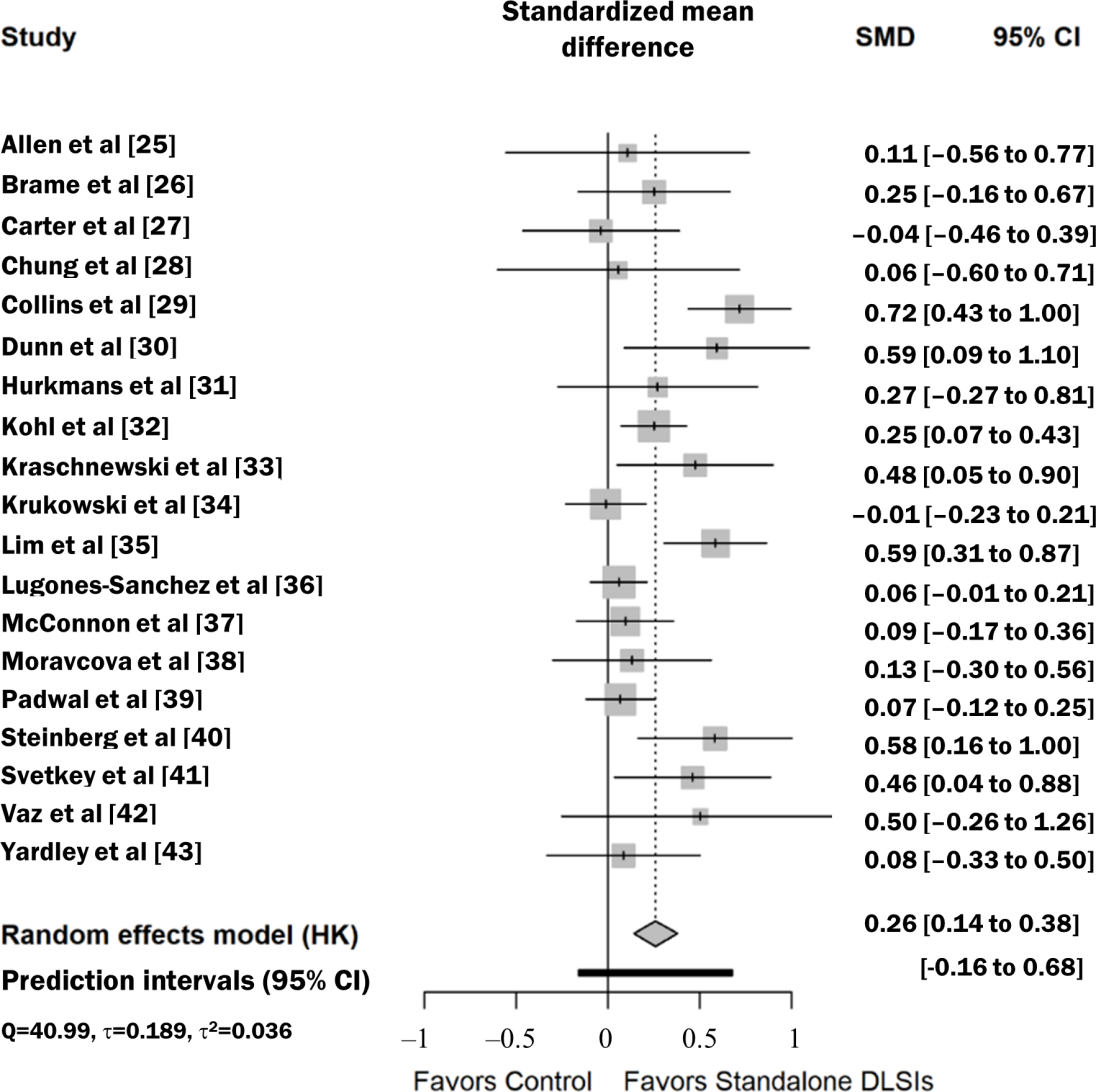
Effect of stand-alone digital lifestyle interventions (DLSIs) on anthropometric outcomes. Forest plot displaying the effect sizes of digital lifestyle interventions on anthropometric outcomes. Gray squares represent the standardized mean difference (SMD) for individual studies, and horizontal gray lines indicate the 95% CIs. The black diamond at the bottom represents the pooled effect size, calculated using the Hartung-Knapp-Sidik-Jonkman (HKSJ) method. A positive SMD favors stand-alone DLSIs [25-43].

**Figure 4.**
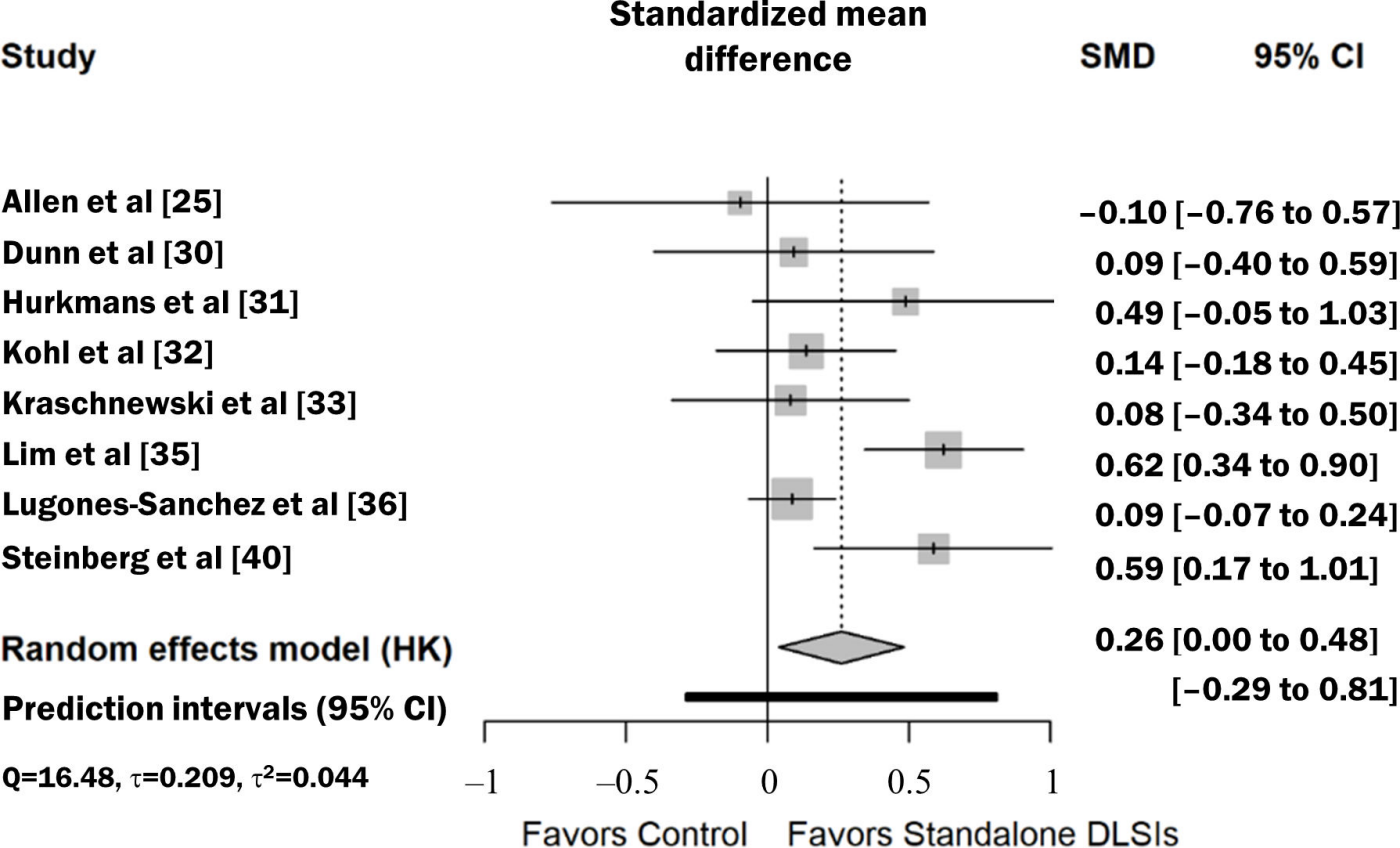
Effect of stand-alone digital lifestyle interventions (DLSIs) on dietary outcomes. Forest plot displaying the effect sizes of digital lifestyle interventions on dietary outcomes. Gray squares represent the standardized mean difference (SMD) for individual studies, and horizontal gray lines indicate the 95% CIs. The black diamond at the bottom represents the pooled effect size, calculated using the Hartung-Knapp-Sidik-Jonkman (HKSJ) method. A positive SMD favors stand-alone DLSIs [25,30-32,35,36,40].

For all 19 included studies (N=3556), the individual effect sizes for anthropometric outcomes ranged from SMD −0.04 to 0.72. Similarly, for the 8 studies reporting dietary outcomes (n=1365), individual SMDs showed a range of −0.10 to 0.62. The summary data for each study, including means, SDs, and sample sizes for both intervention and control groups at baseline and postintervention, are detailed in Table 2. The forest plots (Figure 3 [25-43] and Figure 4 [25,30-32,35,36,40]) provide a visual representation of the individual study effects alongside their 95% CIs.

### Results of Syntheses

The meta-analysis using a random-effects model with the HKSJ adjustment demonstrated that stand-alone DLSIs had a statistically significant effect on improving anthropometric outcomes compared to controls (SMD 0.26, 95% CI 0.14‐0.38; *P*<.001; Table 3). To aid clinical interpretation, the pooled SMD was translated into kilograms based on a pooled baseline SD of 16.7 kg, derived strictly from the included studies that reported body weight in kilograms. This corresponds to an additional weight loss of approximately 4.34 kg. Considering the variability in baseline SDs across the included studies (ranging from 10.1 kg to 25.2 kg), the estimated weight loss could plausibly range from 2.62 kg to 6.55 kg, depending on the population’s baseline variance. While the *I*^2^ statistic (56.1%; *P*<.001) indicated substantial heterogeneity (Q=40.99; *P*<.001; *τ*=0.189; τ^2^=0.036), the 95% PI was calculated as −0.16 to 0.68. This indicates that while the average effect is significant, the outcome in specific future settings is expected to vary, and there is a possibility that the intervention may show no benefit or even be less effective than the control in certain contexts. For dietary outcomes, stand-alone DLSIs also showed a significant effect (SMD 0.26, 95% CI 0.04‐0.48; *P*=.008; Table 3). Substantial heterogeneity (*I*^2^=57.5%; *P*=.01) was observed (Q=16.48; *P*=.01; *τ*=0.209; τ^2^=0.044), and the 95% PI ranged from −0.29 to 0.81, indicating that while the average effect is positive, the impact of these interventions in a new specific setting could vary and may not always achieve a positive result.

**Table 3.** Effects of stand-alone digital lifestyle interventions on anthropometric and dietary outcomes. In cases where a study reported outcomes for multiple intervention arms compared to a single control group, we combined the intervention groups or split the control group to maintain the independence of observations.

Outcomes	Anthropometric outcomes							Dietary outcomes						
Main analysis	*k*	SMD^a^ (95% CI)	95% PI^b^	*I^2^*	Q	τ	τ^2^	*k*	SMD (95% CI)	95% CI	*I^2^*	Q	τ	τ^2^
Overall effects	19	0.26 (0.14 to 0.38)^c^	−0.16 to 0.68	56.1^c^	40.99	0.189	0.036	8	0.26 (0.04 to 0.48)^d^	−0.29 to 0.81	57.5^e^	16.48^e^	0.209	0.044
Subgroup analysis														
Target lifestyle							*P*_subgroup_=.27							—^f^
Diet and physical activity	15	0.27 (0.12 to 0.42)^c^	−0.04 to 0.58	67.5^c^	36.92^c^	0.000	0.000	7	0.30 (0.08 to 0.53)^*^	−0.16 to 0.76	62.6^d^	18.72^d^	0.228	0.052
Diet	4	0.14 (−0.05 to 0.32)	−0.07 to 0.35	0.0	4.31	0.205	0.042	Not applicable as only one study remained	Not applicable as only one study remained	Not applicable as only one study remained	Not applicable as only one study remained	Not applicable as only one study remained	Not applicable as only one study remained	Not applicable as only one study remained
Duration							*P*_subgroup_=.71							*P*_subgroup_=.44
≤12 weeks	10	0.23 (0.07 to 0.40)^d^	−0.17 to 0.65	56.1^d^	20.48^d^	0.165	0.027	5	0.12 (−0.06 to 0.30)	−0.30 to 0.54	0	2.38	0.119	0.014
>12 weeks	9	0.28 (0.06 to 0.50)^e^	−0.27 to 0.84	60.4^d^	20.21^d^	0.221	0.9	3	0.47 (−0.21 to 1.16)	−0.70 to 1.65	42.6	3.48	0.215	0.046
Control group							*P*_subgroup_<.001							—
Active controls	8	0.04 (−0.08 to 0.16)	−0.08 to 0.16	0.00	4.61	0.000	0.000	3	0.23 (−0.23 to 0.70)	−0.46 to 0.92	64.4	5.62	0.324	0.105
Nonspecific active controls	5	0.13 (0.00 to 0.26)^e^	0.00 to 0.26	0.00	2.15	0.000	0.000	Not applicable as only one study remained	Not applicable as only one study remained	Not applicable as only one study remained	Not applicable as only one study remained	Not applicable as only one study remained	Not applicable as only one study remained	Not applicable as only one study remained
Waitlist controls	9	0.57 (0.42 to 0.73)^c^	0.42 to 0.72	0.00	7.91	0.000	0.000	5	0.36 (0.11 to 0.62)^d^	0.11 to 0.61	44.2	7.17	0.145	0.021
Delivery type							*P*_subgroup_=.90							*P*_subgroup_=.61
App	8	0.24 (−0.00 to 0.49)	−0.20 to 0.38	62.3^d^	15.92^e^	0.219	0.048	5	0.33 (0.02 to 0.64)^e^	−0.21 to 0.87	73.6^d^	15.15^d^	0.266	0.071
Website	15	0.23 (0.08 to 0.37)^d^	−0.03 to 0.49	60.2^d^	27.64^d^	0.167	0.028	4	0.22 (−0.04 to 0.48)	−0.04 to 0.48	22.7	3.88	0.089	0.008
Sensitivity analysis														
Aggregated (study-level)	19	0.27 (0.14 to 0.41)^c^	−0.15 to 0.70	56.7^d^	34.65^d^	0.188	0.035	8	0.22 (0.02 to 0.42)^e^	−0.32 to 0.75	54.7^e^	13.25	0.192	0.037
Excluding high risk of bias	16	0.27 (0.14 to 0.41)^c^	−0.21 to 0.74	88.1^c^	126.12^c^	0.213	0.045	7	0.20 (0.00 to 0.39)^e^	−0.37 to 0.77	64.2^e^	16.74	0.211	0.044
≤6-month duration	15	0.26 (0.13 to 0.39)^c^	−0.22 to 0.74	88.1^c^	117.41	0.214	0.046	7	0.26 (0.03 to 0.48)^e^	−0.34 to 0.86	64.3^e^	16.81	0.219	0.048

a SMD: standardized mean difference.

b PI: prediction interval.

c P<.001.

d P<.01.

e *P*<.05.

f Not available.

Subgroup analyses were conducted to explore potential moderators of intervention effects. First, analysis by target behavior revealed that interventions focusing on both diet and physical activity demonstrated a small significant effect on anthropometric outcomes (SMD 0.27, 95% CI 0.12‐0.42; 95% PI −0.04 to 0.58; *P*<.001; *I*^2^=67.5%), while diet-only interventions did not reach statistical significance (SMD 0.14, 95% CI −0.05 to 0.32; 95% PI −0.07 to 0.35; *P*=.12; *I*^2^=0%). For dietary outcomes, combined-target interventions yielded a significant effect (SMD 0.30, 95% CI 0.08‐0.53; 95% PI −0.16 to 0.76; *P*=.008; *I*^2^=62.6%; Table 3).

Second, intervention duration was examined. Stand-alone DLSIs ≤12 weeks showed a small, significant effect on anthropometric outcomes (SMD 0.23, 95% CI 0.07‐0.40; 95% PI −0.17 to 0.65; *P*=.02; *I*^2^=56.1%), comparable to those >12 weeks (SMD 0.28, 95% CI 0.06‐0.50; 95% PI −0.27 to 0.84; *P*=.01; *I*^2^=60.4%). The test for subgroup differences confirmed no significant disparity between short- and long-term durations (*P*=.71). For dietary outcomes, longer interventions did not show a significant effect (SMD 0.47, 95% CI −0.21 to 1.16; 95% PI −0.70 to 1.65; *P*>.05; *I*^2^=42.6%), although no significant heterogeneity was observed. Similarly, shorter ones did not reach significance (SMD 0.12, 95% CI −0.06‐0.30; 95% PI −0.30 to 0.54; *P*>.05; *I*^2^=0%), despite having no heterogeneity (Table 3). The formal test for subgroup differences indicated that the difference between these 2 duration categories was not statistically significant (*P*=.44). This suggests that intervention duration does not significantly moderate the effects on dietary outcomes, and stand-alone DLSIs did not yield a consistent significant impact on diet regardless of the program length.

Third, by control type, stand-alone DLSIs showed no significant effects vs active controls on anthropometric outcomes (SMD 0.04, 95% CI −0.08 to 0.16; 95% PI −0.08 to 0.16; *P*=.52; *I*^2^=0.0%) and diet (SMD 0.23, 95% CI −0.23‐0.70; 95% PI −0.46 to 0.92; *P*=.33; *I*^2^=64.4%). Compared to nonspecific active controls, the effect on anthropometric outcomes was small but significant (SMD 0.13, 95% CI 0.00‐0.26; 95% PI 0.00 to 0.26; *P*=.05; *I*^2^=0%). The strongest effects were observed vs waitlist controls, with a moderate effect on anthropometric outcomes (SMD 0.57, 95% CI 0.42‐0.73; 95% PI 0.42 to 0.72; *P*<.001; *I*^2^=0%) and a significant effect on diet (SMD 0.36, 95% CI 0.11‐0.62; 95% PI 0.11 to 0.61; *P*=.006; *I*^2^=44.2%). A significant subgroup difference was observed for anthropometric outcomes (*P*<.001), indicating control type as a significant moderator (Table 3).

Fourth, by delivery type, interventions delivered via mobile apps showed no significant effect on anthropometric outcomes (SMD 0.24, 95% CI 0.00‐0.49; 95% PI −0.20 to 0.68; *P*=.05; *I*^2^=62.3%), whereas a significant effect was observed for dietary outcomes (SMD 0.33, 95% CI 0.02‐0.64; 95% PI −0.21 to 0.87; *P*=.03; *I*^2^=73.6%). Website-based interventions also demonstrated a significant effect on anthropometric outcomes (SMD 0.23, 95% CI 0.08‐0.37; 95% PI −0.03 to 0.49; *P*=.004; *I*^2^=60.2%), but did not reach statistical significance for dietary outcomes (SMD 0.22, 95% CI −0.04‐0.48; 95% PI −0.04 to 0.48; *P*=.10; *I*^2^=22.7%; Table 3). No significant subgroup differences were found between app and website delivery for either anthropometric (*P*=.90) or dietary outcomes (*P*=.61; Table 3).

Sensitivity analyses were conducted by aggregating multiple effect sizes within the same study using their arithmetic average to evaluate the robustness of the primary findings against potential unit-of-analysis errors. The significant effects of stand-alone DLSIs on both anthropometric and dietary outcomes remained robust. For anthropometric outcomes, the pooled effect size slightly increased (SMD 0.27, 95% CI 0.14‐0.41; *P*<.001; 95% PI −0.15 to 0.70; *I*^2^=56.7%), while for dietary outcomes, the effect size decreased modestly (SMD 0.22, 95% CI 0.02‐0.42; *P*=.03; 95% PI −0.32 to 0.75; I^2^=54.7%; Table 3). In addition, a secondary sensitivity analysis was performed by excluding 3 studies identified as having a high risk of bias while maintaining the study-level aggregation of effect sizes. For anthropometric outcomes, the pooled effect size remained statistically significant (SMD 0.27, 95% CI 0.14‐0.39; *P*<.001; 95% PI −0.21 to 0.74; *I*^2^=88.1%). Similarly, for dietary outcomes, the effect remained significant even after excluding lower-quality evidence (SMD 0.20, 95% CI 0.01‐0.39; *P*=.04; 95% PI −0.37 to 0.77; *I*^2^=64.2%).

Furthermore, to ensure the findings were not skewed by the inclusion of long-term trials, a third sensitivity analysis was restricted to trials with a duration of ≤6 months. For anthropometric outcomes, the effect remained highly consistent (SMD 0.26, 95% CI 0.13‐0.39; *P*<.001; 95% PI −0.22 to 0.74; *I*^2^=88.1%). Similarly, for dietary outcomes, the significant benefit was maintained (SMD 0.26, 95% CI 0.04‐0.48; *P*=.03; 95% PI −0.34 to 0.86; *I*^2^=64.3%; Table 3). These cumulative results indicate that the overall conclusions were not sensitive to the presence of multiple outcomes per study, the inclusion of studies with a high risk of bias, or variations in trial duration, further confirming the stability and reliability of the main analysis.

### Reporting Biases

**Figure 5.**
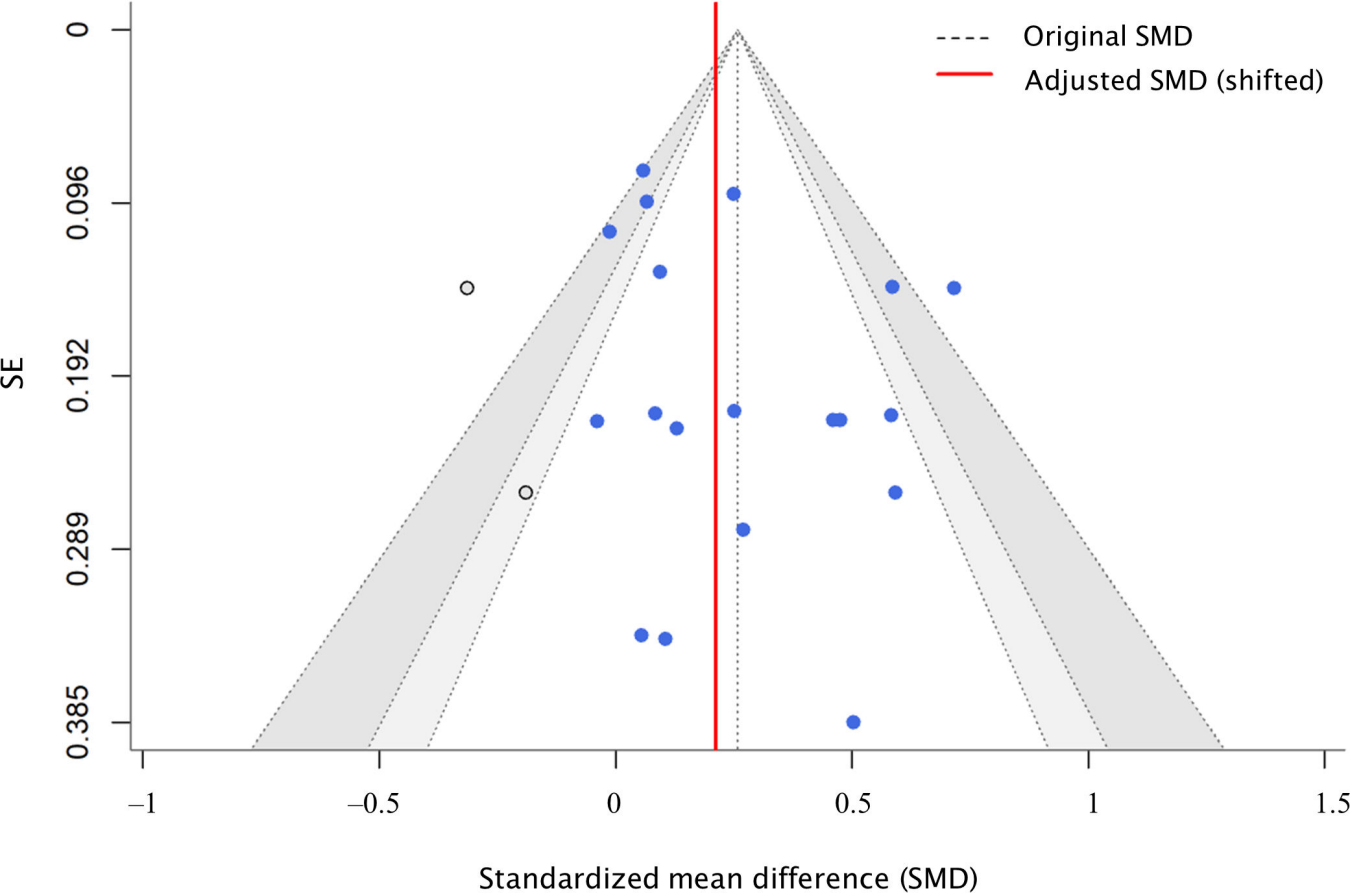
Trim-and-fill funnel plot for anthropometric outcomes. Open circles indicate 2 imputed studies identified via trim-and-fill analysis to address visual asymmetry. The model uses the Hartung-Knapp-Sidik-Jonkman (HKSJ) adjustment. The pooled effect remains statistically significant after imputation, confirming the robustness of the anthropometric results against potential publication bias.

Small-study effects were assessed for significant results in the main analysis using funnel plots and the Egger test. For anthropometric outcomes, the Egger test yielded an intercept of 1.35 (95% CI –1.54 to 4.24; *t*_17_=0.946; *P*=.35), and for dietary outcomes, the intercept was 0.28 (95% CI –1.19 to 1.75; *t*_6_=0.108; *P*=.92), indicating no statistical evidence of asymmetry in either case (Figures5  6). However, specifically for anthropometric outcomes, visual inspection suggested potential asymmetry in the lower-left quadrant, possibly due to the heavy reliance on waitlist-controlled trials and the risk of unpublished small-scale trials. To address this, a trim-and-fill sensitivity analysis was performed (Figure 5), which identified 2 potentially missing studies on the left side of the funnel. After imputing these studies, the adjusted pooled effect size remained statistically significant (adjusted SMD 0.21, 95% CI: 0.09‐0.34; *P*<.001). The heterogeneity for the adjusted model was observed (Q_20_=120.54; *P*<.001; *τ*=0.294; τ^2^=0.086), confirming the robustness of the primary findings. Consequently, the observed effect sizes for both outcomes are unlikely to be driven primarily by publication bias.

**Figure 6.**
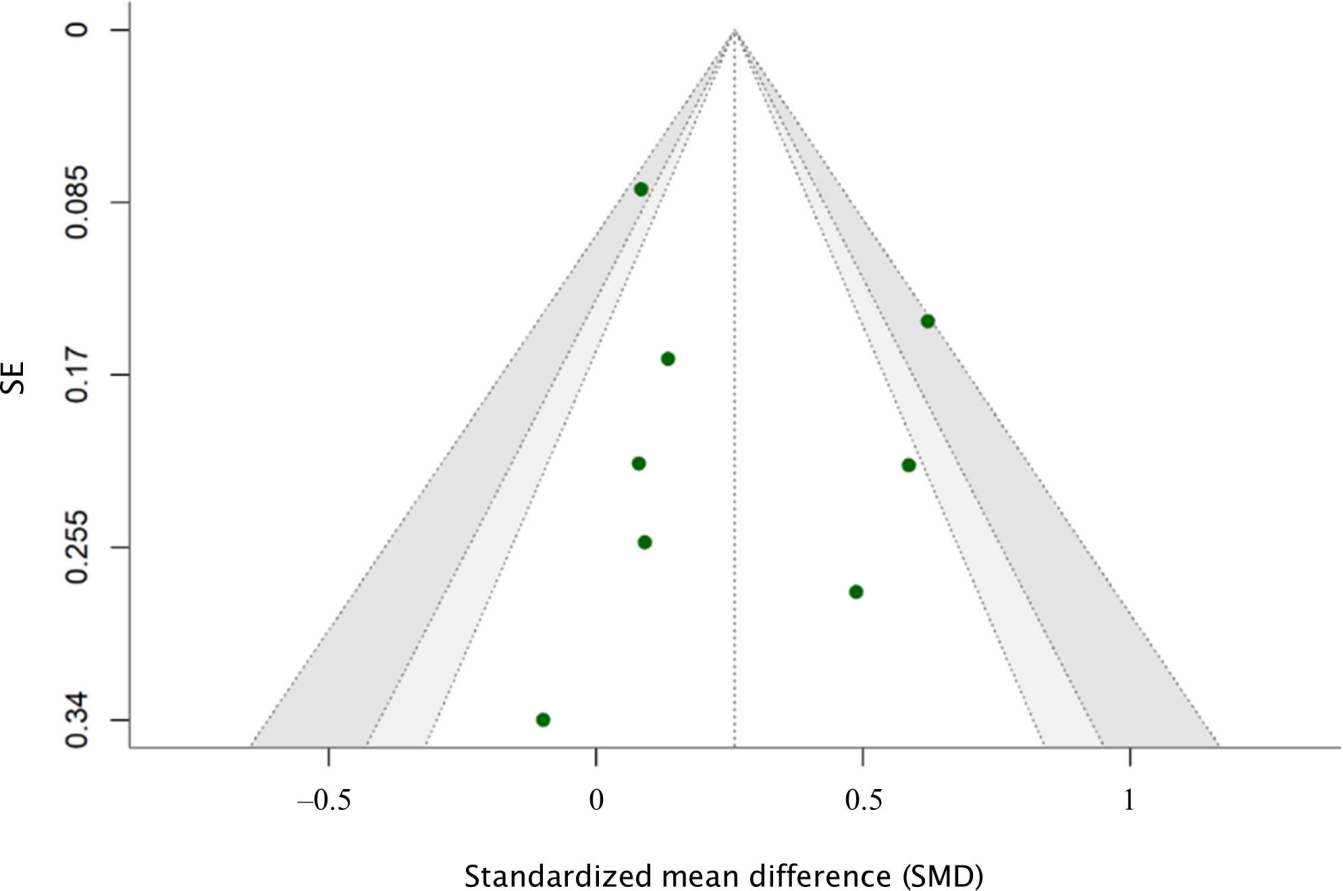
Funnel plot for dietary outcomes. The funnel plot illustrates the relationship between standardized mean differences (SMDs) and their corresponding SEs for dietary outcomes. The plot was generated using the Hartung-Knapp-Sidik-Jonkman (HKSJ) adjustment to account for study heterogeneity.

### Certainty of Evidence

Based on the Grading of Recommendations, Assessment, Development, and Evaluation assessment of each significant effect estimate, this study concludes that stand-alone DLSIs provide a moderate-certainty benefit for both anthropometric and dietary outcomes. The certainty of evidence for anthropometric outcomes was downgraded by one level due to serious concerns regarding inconsistency. This decision was made because of the substantial statistical heterogeneity. Furthermore, our subgroup analysis revealed that the type of control group was a key source of heterogeneity, indicating that the observed dispersion was largely explained by variations in study design rather than inconsistent intervention effects. Similarly, the certainty of evidence for dietary outcomes was downgraded by one level due to serious inconsistency among studies, despite a consistent direction of effects (Table 4).

**Table 4.** Summary of Grading of Recommendations, Assessment, Development, and Evaluation (GRADE) assessment for each outcome.

Outcomes	No of studies	Risk of bias	Inconsistency	Indirectness	Imprecision	Other considerations	SMD^a^ (95% CI)	Quality
Anthropometric outcomes	19 (3556 participants)	Not serious	Serious inconsistency	Not serious	Not serious	Not serious	0.26 (0.14‐0.38) (equivalent to 2.62-6.55 kg reduction)	⊕⊕⊕○ Moderate
Dietary outcomes	8 (1365 participants)	Not serious	Serious inconsistency	Not serious	Not serious	Not serious	0.26 (0.04‐0.48)	⊕⊕⊕○ Moderate

a SMD: standardized mean difference.

### Meta-Regression

**Figure 7.**
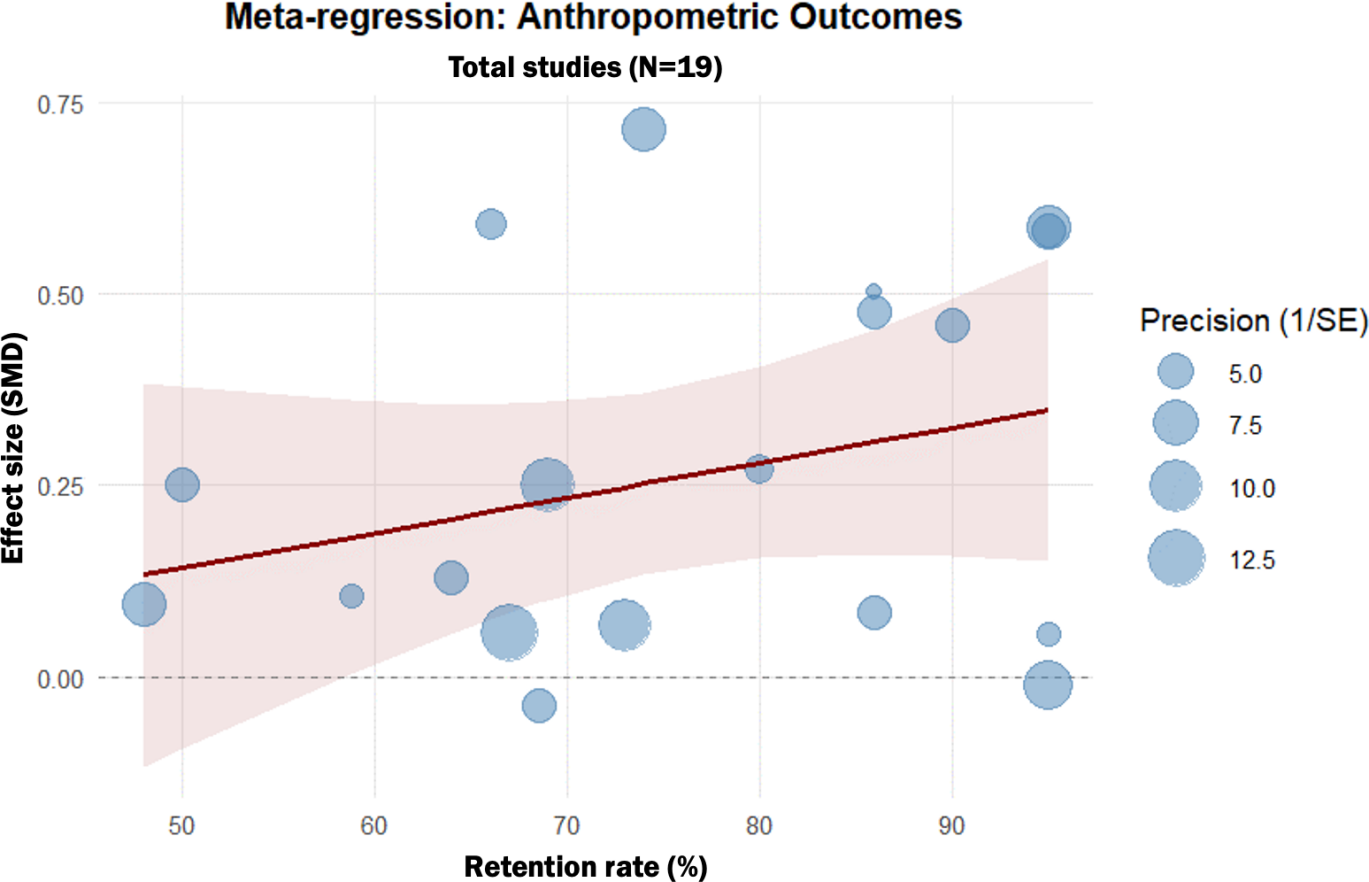
Meta-regression of participant retention rates on anthropometric outcomes. This bubble plot shows the association between the retention rate (%) and the standardized mean difference (SMD) of anthropometric outcomes. Each bubble represents a single study, with the size of the bubble corresponding to the inverse of the variance (study weight). The solid line represents the fitted regression line, showing a significant positive slope.

Meta-regression analysis showed that retention rates impact both anthropometric (β=0.005, 95% CI 0.0001‐0.0100; *P*=.05) and dietary outcomes (β=0.0175, 95% CI 0.0103‐0.0246; *P*<.001), indicating that higher participant retention was associated with greater intervention effectiveness (Figures7  8). This suggests that an increase in participant retention was associated with a corresponding increase in the SMD, highlighting the importance of strategies to minimize dropout in digital interventions.

**Figure 8.**
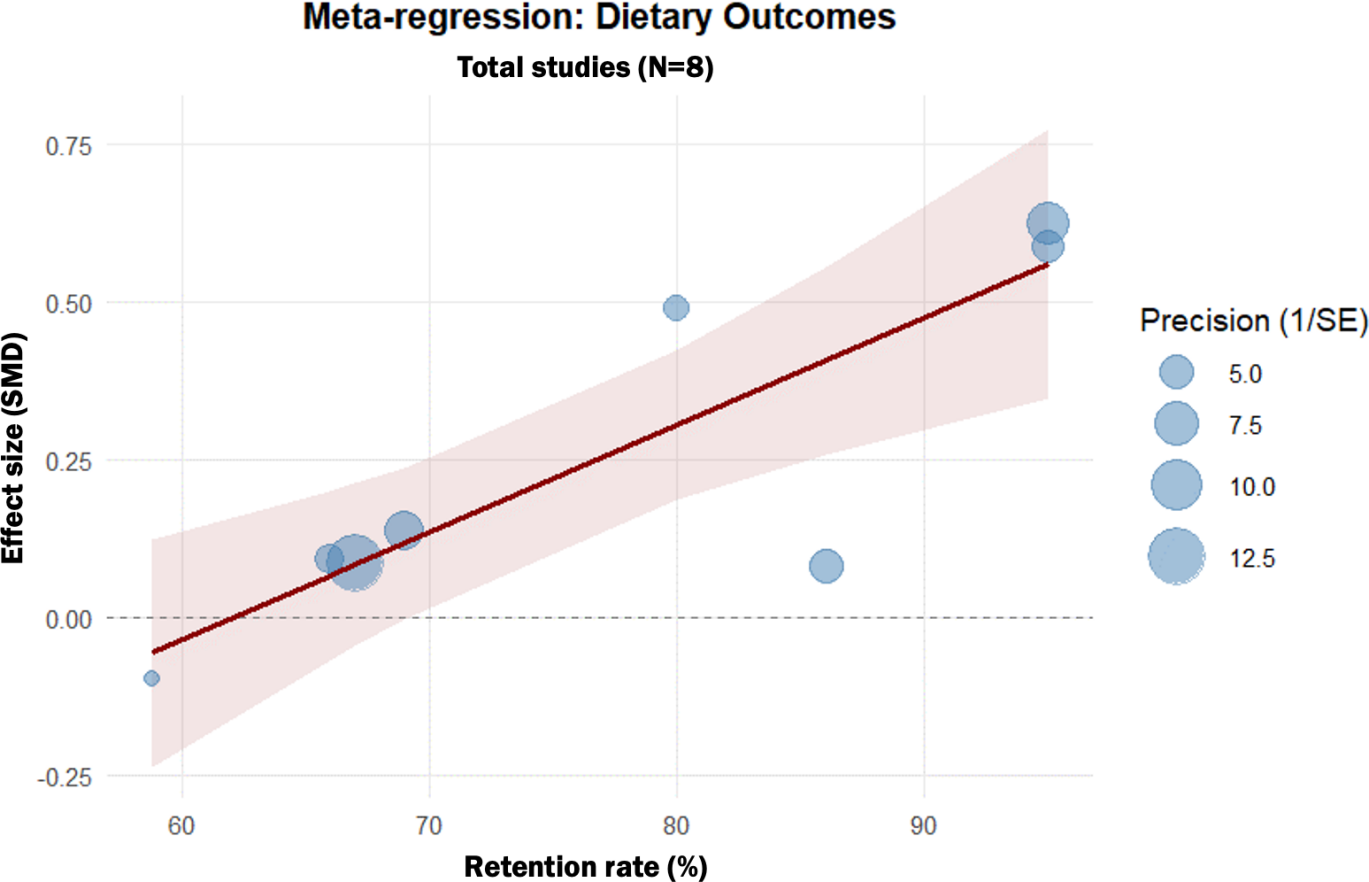
Meta-regression of participant retention rates on dietary outcomes. This bubble plot illustrates the relationship between retention rate (%) and the standardized mean difference (SMD) for dietary outcomes. Each bubble represents a single study, with the size of the bubble corresponding to the inverse of the variance (study weight). The solid line represents the fitted regression line, showing a significant positive slope.

## Discussion

### Interpretation

This systematic review and meta-analysis evaluated the efficacy of stand-alone DLSIs on anthropometric and dietary outcomes among adults with overweight or obesity, with a rigorous focus on isolating the independent therapeutic potential of digital technology. Our findings demonstrate that stand-alone DLSIs can achieve statistically significant improvements in both anthropometric and dietary outcomes, supporting their role as independent interventions. Notably, the observed magnitude of weight loss exceeds the conventional threshold for a minimal clinically important difference in obesity management, which is typically cited as 2‐3 kg [6]. This suggests that stand-alone digital platforms can achieve clinically meaningful outcomes comparable to more resource-intensive traditional programs, reinforcing their viability as a scalable public health tool, especially in resource-constrained settings [44].

To our knowledge, this study is innovative as it is the first to strictly delineate the effect of DLSIs by eliminating adjunctive human interaction. Distinct from previous meta-analyses that often included multimodal or hybrid interventions [14,15], this study clarifies the specific therapeutic potential of digital technology alone. Prior research incorporating structured group formats or peer interactions often reported numerically larger effect sizes [14,15]. However, our results confirm that stand-alone DLSIs still yield statistically significant benefits, suggesting that the digital component itself is a robust driver of change. This reinforces the utility of these interventions in environments where clinical labor or real-time moderation is unavailable [45].

To ensure a balanced interpretation of these results, several analytical factors must be considered. First, the observed statistical heterogeneity reflects the inherent diversity in digital health implementation [46]. Notably, our subgroup analyses clarified that this variance was primarily driven by comparator intensity, as heterogeneity was fully resolved in the active-control subgroup. Interventions targeting both diet and physical activity demonstrated synergistic effects, likely due to more comprehensive behavioral self-regulation [47,48]. Regarding intervention duration, an interesting discrepancy emerged: stand-alone DLSIs demonstrated consistent and significant benefits for anthropometric outcomes regardless of program length, indicating a durable impact on physical parameters. In contrast, dietary outcomes did not show statistically significant improvements in either short-term or long-term subgroups. The lack of significant effect across all timeframes suggests that stand-alone DLSIs may face challenges in independently facilitating meaningful dietary changes, regardless of the duration provided [49,50]. This discrepancy between anthropometric success and dietary stagnation may imply that while digital tools effectively promote weight loss—potentially through enhanced self-monitoring or physical activity tracking—they may require additional integrated support, such as environmental modifications, to overcome the complexities of dietary habit transformation [15]. Furthermore, effectiveness followed a gradient based on comparator type, being most effective against waitlist controls and diminishing against structured active comparators, likely reflecting nonspecific intervention effects such as attention or structure [51,52]. Meta-regression analyses further revealed that higher retention rates were significantly associated with greater improvements in both outcomes, underscoring that sustained user engagement is a critical moderator of effectiveness [51,52].

Furthermore, the risk of bias within the evidence base must be noted, as approximately 63.2% of the included studies were rated as having high risk or some concerns. Sensitivity analyses, however, supported the robustness of these findings, as excluding studies with a high risk of bias preserved the significance of the main results while leading to a marked reduction in heterogeneity, thereby reinforcing the reliability of the observed effects across higher-quality trials.

The credibility of these results is further reflected in the certainty of evidence. The certainty for both anthropometric and dietary outcomes was rated moderate and was primarily downgraded due to serious inconsistency. This moderate certainty indicates that while the current evidence is promising, further high-quality research will be valuable to increase the precision of these estimates and confirm the stability of the observed effects across diverse intervention strategies.

Importantly, the interpretation of these findings requires a clear distinction between the average effect and the distribution of effects across different settings. While the 95% CIs confirm a significant average effect, the calculated 95% PIs for both outcomes cross the null line. This critical distinction suggests that while stand-alone DLSIs are effective on average, the expected effect in a specific future clinical setting may be less certain, with the potential for null or even negative outcomes depending on the implementation context [53].

This study offers several methodological and practical strengths. First, it is the first meta-analysis to isolate the effects of DLSIs, thereby eliminating potential confounding from adjunctive intervention formats. Second, by evaluating both anthropometric and dietary outcomes across diverse delivery modes and comparator types, this study provides a comprehensive and scalable understanding of stand-alone DLSI effectiveness [6]. Third, the inclusion of detailed subgroup and meta-regression analyses helped clarify the conditions under which stand-alone DLSIs are most effective—particularly in relation to targeted behaviors, intervention duration, and comparator group. Fourth, the exclusive inclusion of RCTs, combined with rigorous quality assessments and sensitivity analyses, enhances the methodological credibility of the findings. Finally, the moderate certainty of evidence for both anthropometric and dietary outcomes further underscores the reliability of the observed effects, reinforcing the potential of stand-alone DLSIs as evidence-based tools for obesity management [24].

### Limitations

Nevertheless, several limitations should be acknowledged when interpreting these findings. First, most included trials featured short intervention periods (typically ≤12 weeks), which restrict conclusions regarding the long-term maintenance of weight-related changes. Second, substantial heterogeneity was not consistently explained by device type or behavioral change techniques, suggesting that the variance may stem from unmeasured variables such as individual-level user interaction patterns. Third, the lack of standardized reporting on intervention intensity and cost-effectiveness hinders direct translation into scalable health policy. Fourth, maintaining user engagement remains an inherent challenge in unsupervised digital programs [51]. Fifth, adherence and user engagement—critical determinants of digital intervention success—were underreported or inconsistently measured across trials, precluding formal analysis of these as moderators. Sixth, the presence of potential publication bias for certain outcomes suggests that the observed effect sizes might be slightly overestimated, necessitating a cautious interpretation [22]. Finally, the absence of formal cost-effectiveness evaluations within the included studies limits the translation of findings into scalable health policy recommendations.

To address these limitations, future research should prioritize long-term RCTs that can evaluate the sustainability of DLSI-induced weight changes beyond the typical short-term window. Additionally, more granular analyses are needed to isolate the active components and delivery mechanisms—such as platform type or specific behavioral change techniques—that contribute most significantly to intervention efficacy. Special attention should be given to developing and testing personalized features, such as AI-driven tailoring that adapts to real-time user data, and the cultural adaptation of content to meet the specific needs of diverse ethnic and linguistic populations [54]. Given the underreporting of adherence and engagement metrics in current trials, future studies should integrate standardized measures of user interaction and test the impact of adaptive strategies, including AI-driven personalization, on maintaining user retention and enhancing outcomes. Finally, incorporating cost-effectiveness analyses will provide critical insight for policymakers considering the adoption of stand-alone DLSIs in routine care and public health programming.

### Implications

This systematic review and meta-analysis provides several critical implications for the field of digital health and obesity management. First, in terms of innovation and distinctiveness, our study is unique in that, unlike previous reviews that often conflated purely digital tools with hybrid models involving in-person coaching, it isolated the stand-alone effect of DLSIs without individualized clinical support. This methodological focus distinguishes our review from existing reviews by strictly excluding human-contact confounding factors to demonstrate that digital technology alone can achieve statistically significant weight loss and dietary improvements [14,15]. Second, regarding its contribution to the field, our findings highlight that while the overall effect size of stand-alone interventions may be smaller than that of human-supported ones, they offer a highly scalable and cost-effective solution for public health. This study specifically identifies that stand-alone DLSIs are most effective when compared to minimal-intervention or waitlist controls, providing a clear benchmark for future digital health tool development. Finally, for real-world implications, these results suggest that stand-alone DLSIs can serve as an accessible first-step intervention in a stepped-care model for obesity [55]. For clinicians and policymakers, this means that stand-alone digital platforms can be widely deployed to populations with limited access to intensive in-person counseling, offering a viable strategy to reduce the global burden of obesity-related diseases [1,2].

### Conclusions

Our study validates stand-alone DLSIs as effective primary tools for obesity management, offering moderate-certainty evidence free from direct human-contact confounding. This review contributes to the field by validating technology-driven interventions as effective primary tools. In the real world, these results imply that stand-alone DLSIs offer a scalable first step in stepped-care models, providing an accessible strategy for clinicians and policymakers to address the global obesity burden. However, given the substantial heterogeneity and 95% PIs crossing the null, these average effects should not be viewed as guaranteed benefits. The context-dependent efficacy of stand-alone DLSIs suggests that while they are a viable public health alternative, their implementation requires careful monitoring for consistency across different settings.

## Supplementary material

10.2196/81070 Multimedia Appendix 1 Search strategy.

10.2196/81070 Multimedia Appendix 2 Behavior change techniques (BCTs) applied in the included studies.

10.2196/81070 Multimedia Appendix 3 Verification of digital-led delivery mechanisms and intervention components in the included trials.

10.2196/81070 Checklist 1 PRISMA 2020 checklist.

10.2196/81070 Checklist 2 PRISMA-S checklist.

## Abbreviations

AI: artificial intelligence
BCT: behavior change technique
DLSI: digital lifestyle intervention
HKSJ: Hartung-Knapp-Sidik-Jonkman
PI: prediction interval
PRISMA: Preferred Reporting Items for Systematic Reviews and Meta-Analyses
PRISMA-S: Preferred Reporting Items for Systematic Reviews and Meta-Analyses–Search Extension
RCT: randomized controlled trial
SMD: standardized mean difference
WHO: World Health Organization
