# Supplementary material for: Effects of Stand-Alone Digital Lifestyle Interventions on Weight-Related Outcomes in Adults With Overweight or Obesity: Systematic Review and Meta-Analysis of Randomized Controlled Trials
Source: J Med Internet Res. 2026 May 4;28:e81070. doi: 10.2196/81070 (PMC13139757; doi:10.2196/81070)
Supplement: Multimedia Appendix 1 [file jmir-v28-e81070-s001.docx]

**1. Medline**

1. exp Obesity/

2. exp Overweight/

3. exp Body Weight/

4. exp Weight Loss/

5. exp Weight Reduction Programs/

6. exp Body Mass Index/

7. exp Adiposity/

8. (obesity OR obese OR overweight OR “body weight” OR “weight loss*” OR “weight reduction*” OR “weight management*” OR “weight decrease*” OR “weight control*” OR “weight maintenance*” OR “body mass index” OR BMI or adiposity OR “body fat” OR “adipose tissue” OR “waist circumference*” OR “weight regain prevention” OR “body composition”).ti,ab,kf.

9. 1 OR 2 OR 3 OR 4 OR 5 OR 6 OR 7 OR 8

10. exp Internet-Based Intervention/

11. exp Telemedicine/

12. exp Mobile Applications/

13. exp Text Messaging/

14. exp Smartphone/

15. exp User-Centered Design/

16. (digital OR internet OR online or web* OR mobile OR app OR apps OR smartphone* OR "mobile phone*" OR "cell phone*" OR SMS OR "text message*" OR ehealth OR mhealth OR "tele-health" OR "information technology" OR software OR "digital platform*" OR "fully automated" OR "self-guided" OR standalone OR "self-help" OR "technology-delivered" OR "computer-based" OR automated OR "social media" OR "artificial intelligence" OR "mobile health" OR chatbot* or “conversational agent*” OR wearable* OR “fitness tracker*” OR gamification OR “remote monitoring”).ti,ab,kf.

17. 10 OR 11 OR 12 OR 13 OR 14 OR 15 OR 16

18. randomized controlled trial.pt.

19. controlled clinical trial.pt.

20. randomized.ab.

21. placebo.ab.

22. drug therapy.fs.

23. randomly.ab.

24. trial.ab.

25. groups.ab.

26. 18 OR 19 OR 20 OR 21 OR 22 OR 23 OR 24 OR 25

27. exp animals/ not humans.sh.

28. 26 NOT 27

29. 9 AND 17 AND 28

**2. Embase**

#1 'obesity'/exp

#2 'overweight'/exp

#3 'body weight'/exp

#4 'weight reduction'/exp

#5 'weight control'/exp

#6 'body mass index'/exp

#7 'adipose tissue'/exp

#8 (obesity OR obese OR overweight OR 'body weight' OR ‘weight loss*’ OR 'weight reduction*’ OR ‘weight management*’ OR ‘weight decrease*’ OR ‘weight control*’ OR ‘weight maintenance*’ OR 'body mass index' OR BMI OR adiposity OR 'body fat' OR 'adipose tissue' OR ‘waist circumference*” OR ‘weight remain prevention’ OR ‘body composition’):ti,ab,kw

#9 #1 OR #2 OR #3 OR #4 OR #5 OR #6 OR #7 OR #8

#10 'telemedicine'/exp

#11 'mobile application'/exp

#12 'smartphone'/exp

#13 'text messaging'/exp

#14 'web-based intervention'/exp

#15 'digital health'/exp

#16 'user centered design'/exp

#17 (digital OR internet OR online OR web* OR mobile OR app OR apps OR smartphone* OR 'mobile phone*' OR 'cell phone*' OR SMS OR 'text message* ' OR ehealth OR mhealth OR 'tele-health' OR 'information technology' OR software OR 'digital platform*' OR 'fully automated' OR 'self-guided' OR standalone OR 'self-help' OR 'technology-delivered' OR 'computer-based' OR automated OR 'social media' OR 'artificial intelligence' OR 'mobile health' OR chatbot* OR ‘conversational agent*’ OR wearable* OR ‘fitness tracker*’ OR gamification OR ‘remote monitoring’):ti,ab,kw

#18 #10 OR #11 OR #12 OR #13 OR #14 OR #15 OR #16 OR #17

#19 'randomized controlled trial'/exp

#20 'randomization'/exp

#21 'controlled clinical trial'/exp

#22 'double-blind procedure'/exp

#23 'single-blind procedure'/exp

#24 'crossover procedure'/exp

#25 random*:ti,ab OR placebo*:ti,ab OR 'double-blind*':ti,ab OR 'single-blind*':ti,ab OR assign*:ti,ab OR allocat*:ti,ab OR volunteer*:ti,ab**

#26 #19 OR #20 OR #21 OR #22 OR #23 OR #24 OR #25

#27 'animal'/exp OR 'nonhuman'/exp OR 'animal experiment'/exp NOT 'human'/exp

#28 #26 NOT #27

#29 #9 AND #18 AND #28

**3. Cochrane Library (CENTRAL)**

#1 MeSH descriptor: [Obesity] explode all trees

#2 MeSH descriptor: [Overweight] explode all trees

#3 MeSH descriptor: [Body Weight] explode all trees

#4 MeSH descriptor: [Weight Loss] explode all trees

#5MeSH descriptor: [Weight Reduction Programs] explode all trees

#6 MeSH descriptor: [Body Mass Index] explode all trees

#7 MeSH descriptor: [Adiposity] explode all trees

#8 (obesity OR obese OR overweight OR "body weight" OR “weight loss*” OR “weight reduction*” OR “weight management*” OR “weight decrease*” OR “weight control*” OR “weight maintenance*” OR "body mass index" or BMI or adiposity OR "body fat" OR “waist circumference*" OR “weight remain prevention” OR “body composition”):ti,ab,kw

#9 #1 OR #2 OR #3 OR #4 OR #5 OR #6 OR #7 OR #8

#10 MeSH descriptor: [Internet-Based Intervention] explode all trees

#11 MeSH descriptor: [Telemedicine] explode all trees

#12 MeSH descriptor: [Mobile Applications] explode all trees

#13 MeSH descriptor: [Text Messaging] explode all trees

#14 MeSH descriptor: [Smartphone] explode all trees

#15 MeSH descriptor: [User-Centered Design] explode all trees

#16 (digital or internet OR online or web* OR mobile OR app OR apps OR smartphone* OR "mobile phone*" OR "cell phone*" OR SMS OR "text message*" OR ehealth or mhealth OR "tele-health" OR "information technology" OR software OR "digital platform*" OR "fully automated" OR "self-guided" OR standalone OR "self-help" OR "technology-delivered" OR "computer-based" OR automated OR "social media" OR "artificial intelligence" OR "mobile health" OR chatbot* OR “conversational agent*” OR wearable* OR “fitness tracker*” OR “remote monitoring”):ti,ab,kw

#17 #10 OR #11 OR #12 OR #13 OR #14 OR #15 OR #16

#18 #9 AND #17

**4. PsycINFO(EBSCOhost)**

S1 (MH "Obesity+") OR (MH "Overweight") OR (MH "Body Weight+") OR (MH "Body Mass Index") OR (MH "Adipose Tissue")

S2 (MH "Weight Loss+") OR (MH "Weight Control+") OR (MH "Weight Measurement+")

S3 TI (obesity OR obese OR overweight OR "body weight" OR “weight loss*” OR “weight reduction*” OR “weight management*” OR “weight decrease*” OR “weight control*” OR “weight maintenance*” OR "body mass index" OR BMI OR adiposity OR "body fat" OR "waist circumference*" OR “weight remain prevention” OR “body composition”)

S4 AB (obesity OR obese OR overweight OR "body weight" OR “weight loss*” OR “weight reduction*” OR “weight management*” OR “weight decrease*” OR “weight control*” OR “weight maintenance*” OR "body mass index" OR BMI OR adiposity OR "body fat" OR "waist circumference*" OR “weight remain prevention” OR “body composition”)

S5 S1 OR S2 OR S3 OR S4

S6 (MH "Internet+") OR (MH "Telemedicine+") OR (MH "Mobile Applications") OR (MH "Text Messaging") OR (MH "Digital Health") OR (MH "User Centered Design")

S7 TI (digital OR internet OR online OR web* OR mobile OR app OR apps OR smartphone* OR "mobile phone*" OR "cell phone*" OR SMS OR "text message*" OR ehealth OR mhealth OR "tele-health" OR "information technology" OR software OR "digital platform" OR "fully automated" OR "self-guided" OR standalone OR "self-help" OR "technology-delivered" OR "computer-based" OR automated OR "social media" OR "artificial intelligence" OR chatbot* OR “conversational agent*” or wearable* OR “fitness tracker*” or gamification OR “remote monitoring”)

S8 AB (digital OR internet OR online OR web* OR mobile OR app OR apps OR smartphone* OR "mobile phone*" OR "cell phone*" OR SMS OR "text message*" OR ehealth OR mhealth OR "tele-health" OR "information technology" OR software OR "digital platform" OR "fully automated" OR "self-guided" OR standalone OR "self-help" OR "technology-delivered" OR "computer-based" OR automated OR "social media" OR "artificial intelligence" OR chatbot* OR “conversational agent*” or wearable* OR “fitness tracker*” or gamification OR “remote monitoring”)

S9 S6 OR S7 OR S8

S10 (MH "Randomized Clinical Trials") OR (MH "Clinical Trials") OR (MH "Placebo")

S11 TI (random* OR placebo* OR "double-blind*" OR "single-blind*" OR assign* OR allocat*) OR AB (random* OR placebo* OR "double-blind*" OR "single-blind*" OR assign* OR allocat*)

S12 S10 OR S11

S13 S5 AND S9 AND S12

**5. Web of Science**

#1 TS=(obesity OR obese OR overweight OR "body weight" OR “weight loss*” OR “weight reduction*” OR “weight management*” OR “weight decrease*” OR “weight control*” OR “weight maintenance*” OR "body mass index" OR BMI OR adiposity OR "body fat" OR "adipose tissue" OR "waist circumference*" OR “weight remain prevention” OR “body composition”)

#2 TS=(digital OR internet OR online OR web* OR mobile OR app OR apps OR smartphone* OR "mobile phone*" OR "cell phone*" OR SMS OR "text message*" OR ehealth OR mhealth OR "tele-health" OR "information technology" OR software OR "digital platform" OR "fully automated" OR "self-guided" OR standalone OR "self-help" OR "technology-delivered" OR "computer-based" OR automated OR "social media" OR "artificial intelligence" OR "mobile health" OR chatbot* OR wearble* OR “fitness tracker*” OR gamification OR “remote monitoring”)

#3 TS=("randomized controlled trial" OR "clinical trial" OR "random allocation" OR "double-blind*" OR "single-blind*" OR "randomly assigned" OR placebo*)

#4 #1 AND #2 AND #3
