# Supplementary material for: Effects of Stand-Alone Digital Lifestyle Interventions on Weight-Related Outcomes in Adults With Overweight or Obesity: Systematic Review and Meta-Analysis of Randomized Controlled Trials
Source: J Med Internet Res. 2026 May 4;28:e81070. doi: 10.2196/81070 (PMC13139757; doi:10.2196/81070)
Supplement: Multimedia Appendix 2 [file jmir-v28-e81070-s002.docx]

| Study (Author, year) | BCT Applied |
| --- | --- |
| Allen et al., 2013 | 1.1 Goal setting (behavior); 2.3 Self-monitoring of behavior; 3.1 Social support (unspecified); 4.1 Instruction on how to perform the behavior; 6.1 Demonstration of behavior; 7.1 Prompts/cues; 8.1 Behavioral practice/rehearsal; 10.4 Social reward; 11.2 Reduce negative emotions; 12.5 Adding objects to the environment; 13.2 Framing/reframing; 15.1 Verbal persuasion about capability; 16.3 Vicarious consequences |
| Brame et al., 2022 | 1.1 Goal setting (behavior); 2.3 Self-monitoring of behavior; 3.1 Social support (unspecified); 4.1 Instruction on how to perform the behavior; 6.1 Demonstration of the behavior; 7.1 Prompts/cues; 10.4 Social reward |
| Carter et al., 2013 | 1.1 Goal setting (behavior); 2.3 Self-monitoring of behavior; 4.1 Instruction on how to perform the behavior; 7.1 Prompts/cues |
| Chung et al., 2014 | 1.1 Goal setting (behavior); 2.3 Self-monitoring of behavior; 3.1 Social support (unspecified); 4.1 Instruction on how to perform the behavior; 7.1 Prompts/cues; 10.4 Social reward |
| Collins et al., 2012 | 1.1 Goal setting (behavior); 2.3 Self-monitoring of behavior; 4.1 Instruction on how to perform the behavior; 6.1 Demonstration of the behavior; 7.1 Prompts/cues |
| Dunn et al., 2016 | 1.1 Goal setting (behavior); 2.3 Self-monitoring of behavior; 3.1 Social support (unspecified); 4.1 Instruction on how to perform the behavior; 5.1 Information about health consequences; 10.4 Social reward |
| Hurkmans et al., 2018 | 1.1 Goal setting (behavior); 2.3 Self-monitoring of behavior; 4.1 Instruction on how to perform the behavior; 7.1 Prompts/cues; 10.4 Social reward |
| Kohl et al., 2023 | 1.1 Goal setting (behavior); 2.3 Self-monitoring of behavior; 4.1 Instruction on how to perform the behavior; 6.1 Demonstration of the behavior; 7.1 Prompts/cues; 10.4 Social reward |
| Krakschnewski et al., 2011 | 1.1 Goal setting (behavior); 2.3 Self-monitoring of behavior; 3.1 Social support (unspecified); 4.1 Instruction on how to perform the behavior; 5.1 Information about health consequences; 10.4 Social reward |
| Krukowski et al., 2011 | 1.1 Goal setting (behavior); 2.3 Self-monitoring of behavior; 4.1 Instruction on how to perform the behavior; 5.1 Information about health consequences; 10.4 Social reward |
| Lim et al., 2021 | 1.1 Goal setting (behavior); 2.3 Self-monitoring of behavior; 4.1 Instruction on how to perform the behavior; 7.1 Prompts/cues |
| Lugones-Sanchez et al., 2022 | 1.1 Goal setting (behavior); 2.3 Self-monitoring of behavior; 4.1 Instruction on how to perform the behavior; 7.1 Prompts/cues; 10.4 Social reward |
| McConnon et al., 2007 | 1.1 Goal setting (behavior); 2.3 Self-monitoring of behavior; 3.1 Social support (unspecified); 4.1 Instruction on how to perform the behavior; 10.4 Social reward |
| Moravcová et al., 2024 | 1.1 Goal setting (behavior); 2.3 Self-monitoring of behavior; 4.1 Instruction on how to perform the behavior; 7.1 Prompts/cues |
| Padwal et al., 2017 | 1.1 Goal setting (behavior); 2.3 Self-monitoring of behavior; 3.1 Social support (unspecified); 4.1 Instruction on how to perform the behavior; 10.4 Social reward |
| Steinberg et al., 2013 | 2.3 Self-monitoring of behavior; 10.4 Social reward |
| Svetkey et al., 2015 | 1.1 Goal setting (behavior); 2.3 Self-monitoring of behavior; 4.1 Instruction on how to perform the behavior; 7.1 Prompts/cues |
| Vaz et al., 2021 | 1.1 Goal setting (behavior); 2.3 Self-monitoring of behavior; 4.1 Instruction on how to perform the behavior; 6.1 Demonstration of the behavior; 7.1 Prompts/cues; 10.4 Social reward |
| Yardley et al., 2014 | 1.1 Goal setting (behavior); 2.3 Self-monitoring of behavior; 3.1 Social support (unspecified); 4.1 Instruction on how to perform the behavior; 7.1 Prompts/cues; 10.4 Social reward |
