# Supplementary material for: Effects of Stand-Alone Digital Lifestyle Interventions on Weight-Related Outcomes in Adults With Overweight or Obesity: Systematic Review and Meta-Analysis of Randomized Controlled Trials
Source: J Med Internet Res. 2026 May 4;28:e81070. doi: 10.2196/81070 (PMC13139757; doi:10.2196/81070)
Supplement: Multimedia Appendix 3 [file jmir-v28-e81070-s003.docx]

| Study (Author, year) | Ambiguous component | Original trial wording | Verification source | Final classification |
| --- | --- | --- | --- | --- |
| Allen et al., 2013^21^ | Behavioral counseling | "Participants in the smartphone-only group received one session of basic nutrition counseling and training in the smartphone application... The system used the Mifflin equation for calculating resting metabolic rate... Instant, real-time calculation of current energy balance allowed the participant to keep on track." | Protocol | Fully automated |
| Brame et al., 2022^22^ | Structured coaching and feedback | "With an interactive, self-directed, and flexible usability, this program enables personalized health coaching according to the users’ health profiles... One preset daily activity was the nutritional protocol... It provided a helpful management tool on energy density, calorie balance, and fluid intake... includes daily tips, notifications, and support opportunities." | Protocol | Fully automated |
| Carter et al., 2013^23^ | Not applicable |  |  |  |
| Chung et al., 2014^24^ | Not applicable |  |  |  |
| Collins et al., 2012^25^ | Not applicable |  |  |  |
| Dunn et al., 2016^26^ | Structured coaching | "The program consisted of 15 lessons... focused on eating and physical activity behaviors... The instructor used best practice techniques for the online environment... including engaging the learner in interaction." | Protocol | Human-involved (standardized one-to-many education) |
| Hurkmans et al., 2018^27^ | Not applicable |  |  |  |
| Kohl et al., 2023^28^ | Not applicable |  |  |  |
| Kraschnewski, 2011^29^ | Tailored behavior modeling | "Algorithms matched participants to three role models... Participants were prompted to build a weight-loss plan by selecting their preferred practices... At each log-in, participants received tailored feedback to help them choose which practices to sustain or adapt, based on their own practice use." | Protocol | Fully automated |
| Krukowski et al., 201130 | Not applicable |  |  |  |
| Lim et al., 202131 | Dietitian messaging | “Participants... were encouraged to achieve individualized calorie and carbohydrate goals... set by the app. Educational videos... were pushed to the participants weekly via the app. The 2 dietitians... supported the participants by messaging them via the app... spending 1 to 15 minutes on each participant each time." | Protocol | Human-involved (asynchronous messaging) |
| Lugones-Sanchez et al., 2022^32^ | Not applicable |  |  |  |
| McConnon et al., 2007^33^ | Not applicable |  |  |  |
| Moravcova et al., 2024^34^ | Dietitian support | "The application uses a series of personalized daily tasks and automated messages to help patients establish a new, healthy routine... a qualified dietitian was available on the in-app to answer participants’ queries or concerns." | Protocol | Human-involved (asynchronous feedback) |
| Padwal et al., 2017^35^ | Not applicable |  |  |  |
| Steinberg et al., 2013^36^ | Behavioral lessons | "An algorithm was used to provide tailored feedback to each participant... Participants were placed in one of four categories each week... They received messages and recommended strategies appropriate for that category. The weekly e-mailed lessons... provided further skills training." | Protocol | Fully Automated |
| Svetkey et al., 2015^37^ | Peer support | "Social support through a 'buddy system' that allowed exchange of pre-determined messages to a randomly assigned buddy participant... Intervention was delivered exclusively through an investigator-designed smartphone app." | Protocol | Fully Automated |
| Vaz et al., 2021^38^ | Coaching and peer feedback | "The professional coach throughout the study was an Obesity Medicine Board‐certified Endocrinologist... Remote professional coaching by the physician consisted of feedback delivered via group and private messaging using shared activity data, shared food photography logs, and daily weights." | Protocol | Human-involved (asynchronous feedback) |
| Yardley et al., 2014^39^ | Not applicable |  |  |  |
| Standalone DLSIs were defined as interventions where therapeutic processes are managed primarily by the digital platform. "Fully Automated" refers to interventions driven exclusively by pre-programmed algorithms or system-generated logic. "Human-involved" refers to models where any human participation is limited to broad educational delivery or asynchronous feedback, without providing resource-intensive, one-to-one clinical counseling. Verification was performed by cross-referencing the technical Implementation described in the Methods section with the original Study Protocols and Trial Registrations. | | | | |
